# Understanding decision making factors influencing referrals to vision rehabilitation services at a tertiary medical center

**DOI:** 10.1371/journal.pone.0359154

**Published:** 2026-10-26

**Authors:** Sanbrita Mondal, Kyle D. Peterson, Bonnie J. Verges, Christina L. Thomas-Virnig

**Affiliations:** Department of Ophthalmology and Visual Sciences, School of Medicine and Public Health, University of Wisconsin, Madison, Wisconsin, United States of America; PLOS: Public Library of Science, UNITED KINGDOM OF GREAT BRITAIN AND NORTHERN IRELAND

## Abstract

Functional vision loss at any stage affects quality of life even before clinically quantified to be moderate or severe. Eye care providers as the gatekeepers to vision rehabilitation services often underestimate how small variations in functional vision loss can have a significant impact in a patient’s life. Greater provider-patient communication about vision loss is an opportunity to build trust and increase access to vision rehabilitation services to improve quality of life. Tertiary eye care centers serve the greatest number of visually impaired patients. The purpose of this study was to find what factors influenced eye care providers with vision rehabilitation service referral decision-making at a tertiary medical center. We also further studied whether decision making was influenced by the eye care provider’s specialty, volume of practice, years of practice, number of practicing locations, gender and continuing education status. Our survey found that about 77% of respondents were early (<10 years) in their career and 75% of posterior segment specialists agreed that people with any functional vision complaint should be referred to services regardless of visual acuity. Fifty-two percent of all respondents indicated the reason for low referrals to services was due to a lack of inquiry from patients. Inquiry about driving status by patients was an important factor for referral to services for 35% of respondents. Forty-five percent of respondents reported lack of vision rehabilitation providers and long wait times as a barrier to referrals. However, only 61% of respondents reported “never” to “sometimes” referring to community services. Posterior specialists attended more continuing education courses related to low vision/ vision rehabilitation than anterior segment specialists. By understanding influencers on eye care providers decision making, we will be able to make more targeted recommendations on workflow changes, continuing education and quality improvement processes to increase access to vision rehabilitation services.

## Introduction

Functional vision loss at any stage affects quality of life (QOL) even before the loss is clinically quantified to be moderate or severe. With decline in functional vision, the patient’s ability to perform social and physical tasks becomes impaired, leading to dependency on others and affecting emotional wellbeing [1]. Vision impairment has a significant health and economic impact not just on the patient, but on their families, society, and the healthcare system [2]. Vision impairment influences physical, cognitive, psychological, social functioning, independence and can be an economic and societal burden [3–5]. With an aging population and an increase in frequency of eye diseases, it is projected the population with vision impairment will double by 2050 [6]. Timely referral to vision rehabilitation services (VRS) is key to maintaining good health related QOL and reduce functional disability caused by vision loss [7,8]. Several studies have demonstrated the effectiveness of VRS towards improving clinical and functional abilities as well as QOL particularly in reading, mobility and well-being [9–11]. Eye care providers (ECP) are the gatekeepers to VRS [12–14]. Referral by ECPs when a patient has a functional vision complaint in relation to their eye disease regardless of visual acuity or visual field status is standard of care [15,16]. Despite this, it is suspected that ECPs mostly rely on patients to inquire about VRS before a referral is made [11,17]. The existence and benefits of VRS are not generally known by the public population [17–20]. This may be a reason why despite the number of patients seen with irreversible vision loss in a tertiary medical center, referral rates remain low [8,11,21].

ECPs often underestimate how small variations in functional vision loss can have a significant impact in a patient’s activities of daily living, vocation, independence, and social life [22–24]. This disparity may be due to the clinic setting where visual outcomes focus on high contrast optotype measurements. The American Academy of Ophthalmology Preferred Practice Patterns (AAO PPP) standard of care guideline recommends referring patients to VRS at any visual acuity when the patient complains their vision impairment affects any activities of daily living. A referral to VRS only when a decline in visual acuity is objectively measured is often too late. The 10^th^ and 11^th^ versions of the International Classification of Disease and Related Health Problems (ICD-10, ICD-11) define low vision as starting at visual acuity worse than 20/70. By this stage, a patient will already have subjective vision impairment affecting activities of daily living requiring greater levels of instrumental support from families [8,24,25]. Patients with visual acuity worse than 20/40 report a greater decrease in their health-related QOL [7]. Significantly, a minimum of 20/40 visual acuity is one of the requirements for an unrestricted driving license in most states in the United States along with visual field requirements [24,26]. Thus, visual acuity parameters for disease diagnosis codes are often not reliable methods for patient referral to VRS.

Due to the limited time spent in clinic with patients and a focus on eye health, effects of vision loss on activities of daily living are often unfortunately missed. This leaves the visually impaired patient with the perception that nothing further can be done to address their daily life struggles except managing eye disease. These patients silently lose their sense of independence while they wait for the latest drug or cure [27,28]. Since often no options are provided to help improve daily life struggles, the trust between a patient and their ECP can erode resulting in treatment non-compliance and/or missed appointments. A worsening of the eye disease may result which could have been easily prevented or managed with better communicative care [29]. Greater patient-provider communication about vision loss is an opportunity to build trust and increase access to VRS to help cope with vision loss as well as address these struggles [30].

Tertiary eye care centers such as hospitals and university-based practices serve the greatest number of visually impaired patients [31] given most of their patients are referred to advanced care due to suffering from uncurable eye diseases. Thus, it is important to identify barriers to care to improve information access and reach a greater population in need. Understanding ECPs’ perception towards vision impairment and VRS referral can help address barriers so those suffering with vision impairment are provided with timely VRS, social work service and behavioral health interventions [3,32].

The purpose of our study was to estimate which factors influenced ECPs with VRS referral decision-making at a tertiary medical center. Here we piloted a survey targeting optometrists and ophthalmologists at a major academic medical center to understand whether decision making was influenced by the ECP’s specialty, volume of practice, years of practice, number of practicing locations, gender and continuing education status. By understanding influences on ECPs decision making, we will be able to make more targeted recommendations on workflow changes, continuing education and quality improvement processes to increase access to care bringing VRS forward as standard of care [15].

## Methods

We surveyed optometrists and ophthalmologists involved with direct patient care at a major academic medical center. Twenty-five questions in the form of multiple choice and Likert scale were prepared (1–5 scale) and reviewed by the University of Wisconsin Survey Center to ensure appropriateness and quality. These questions encompassed provider assessment of functional vision loss, awareness of vision rehabilitation services, driving ability, status of legal blindness, mobility, transportation needs, psychosocial impact and other QOL outcomes (Appendix 1). This report focused on understanding the provider’s influence on referrals to vision rehabilitation services. The psychosocial impact portion of the survey is an under reported issue that will be addressed in a separate focused report given the depth of our findings. All appropriate approvals were obtained through the University of Wisconsin Minimal Risk Research Institutional Review Board (IRB) prior to proceeding with the project. The IRB deemed the activity as not research involving human subjects as defined by Department of Health and Human Services and Food and Drug Administration regulations (Reference UW IRB ID#2023–0119). The information sheet for survey participation had language indicating implied consent. Surveys were stored and conducted using the Qualtrics Survey Hosting Service available at the institute. Invitations to participate in surveys were sent through the institute e-mail server on April 6, 2023. Reminders were made three times after a two-week period to ensure maximum participation. Surveys were sent to 9 optometrists and 30 ophthalmologists actively practicing at the Institute’s outpatient eye clinics. The survey was closed on July 14, 2023. Over the period of three months, we received a total of 34 responses, of which 3 were incomplete. Data collected from responses were accessed for analysis on August 10, 2023. The corresponding author and study coordinator had access to information that could identify individual participants during data collection but not during analysis. Responses were classified based on specialty (optometry (OD) vs. ophthalmology (MD)), years of practice (YOP), number of locations (1–2 vs. 3 or more), gender, practice volume (<30 patients vs. > 30 patients per day), and segment of the eye the respondent specialized in (anterior (AS) vs. posterior (PS) vs. whole eye (WE)). AS specialists are those that treat diseases related to the front of the eye including the cornea, anterior chamber and physiologic lens. PS specialists are those that treat diseases related to the back of the eye including vitreous, retina, choroid and optic nerve. WE specialists are defined as those treating the whole eye health including comprehensive ophthalmologists and optometrists.

For all data analyses, inverse-probability weighing was applied to adjust for stratified sampling between specialties – OD vs. MD and AS vs. PS vs. WE specialties. The finite population correction was used to adjust the variance as a substantial proportion of the total population of interest was sampled. Chi-squared analyses were performed to assess the relationship between categorical responses and specialty. The Rao-Scott second-order correction and the Satterthwaite degrees of freedom approximation were applied to properly adjust for weighting and stratification from the survey sampling scheme. Two sample *t*-tests were performed to assess if integer Likert scale responses differed by specialty, using the survey design-based degrees of freedom and Horvitz-Thompson standard errors. Inferential estimates are reported as mean ± 95% confidence intervals.

## Results

Twenty-four of the 31 completed surveys were from ophthalmologists. The remaining 7 were from optometrists (Table 1). Of these respondents, 58% identified as female. Fifty-five percent of respondents identified themselves as WE specialists. Forty-two percent of respondents were early on in their career with less than 10 YOP.

**Table 1:** Participant demographics. Breakdown of provider responses based on gender, specialty, years of practice, professional degree and number of practice locations.

	N = 31 (100%)
**Gender**	
Female	18 (58%)
Male	13 (42%)
**Specialty**	
Anterior (AS)	6 (19%)
Posterior (PS)	8 (26%)
Both (WE)	17 (55%)
**Years of Practice**	
< 10	13 (42%)
10-19	9 (29%)
20-29	5 (16%)
≥ 30	4 (13%)
**Professional Degree**	
OD	7 (23%)
MD	24 (77%)
**Practice Locations**	
1	11 (35%)
2	11 (35%)
3	7 (23%)
>3	2 (7%)

Data from the survey results showed 76.9% (CI 50.7% − 92.2%) of respondents with less than 10 YOP reported patients should be referred to VRS at any visual acuity compared to 33.3% (CI 11.4% – 64.3%) of respondents with 10–19 YOP and to 44.4% (CI 18.5% − 73.4%) of respondents with 20 or more YOP (Fig 1).Seventy-five percent (CI 41%−94%) of PS specialists reported patients should be referred to VRS at any visual acuity vs. 50% (CI 22%−78%) of AS specialists and 47% (CI 27%−68%) of respondents who specialize in WE (Fig 2). There was no significant difference in response based on gender or location.

**Fig 1:**
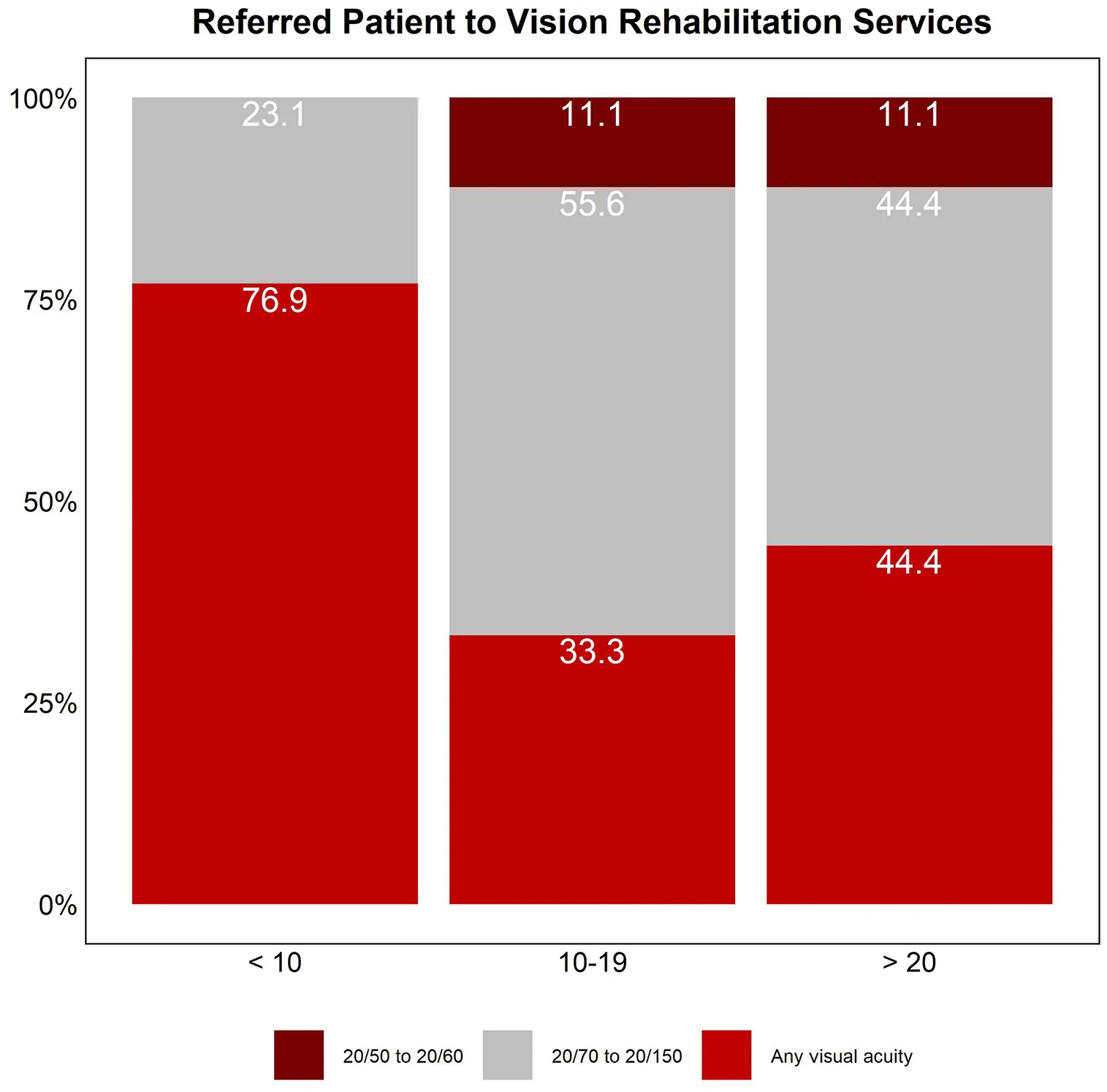
Referred patients to VRS. Proportion of referred patients to VRS stratified by practitioner years of practice (categories: < 10, 10-19, > 20). Stacked bars and values represent the mean proportion of patients within each visual acuity category: 20/50 to 20/60 (dark red), 20/70 to 20/150 (gray), and any visual acuity (red). Confidence intervals are omitted for clarity.

**Fig 2:**
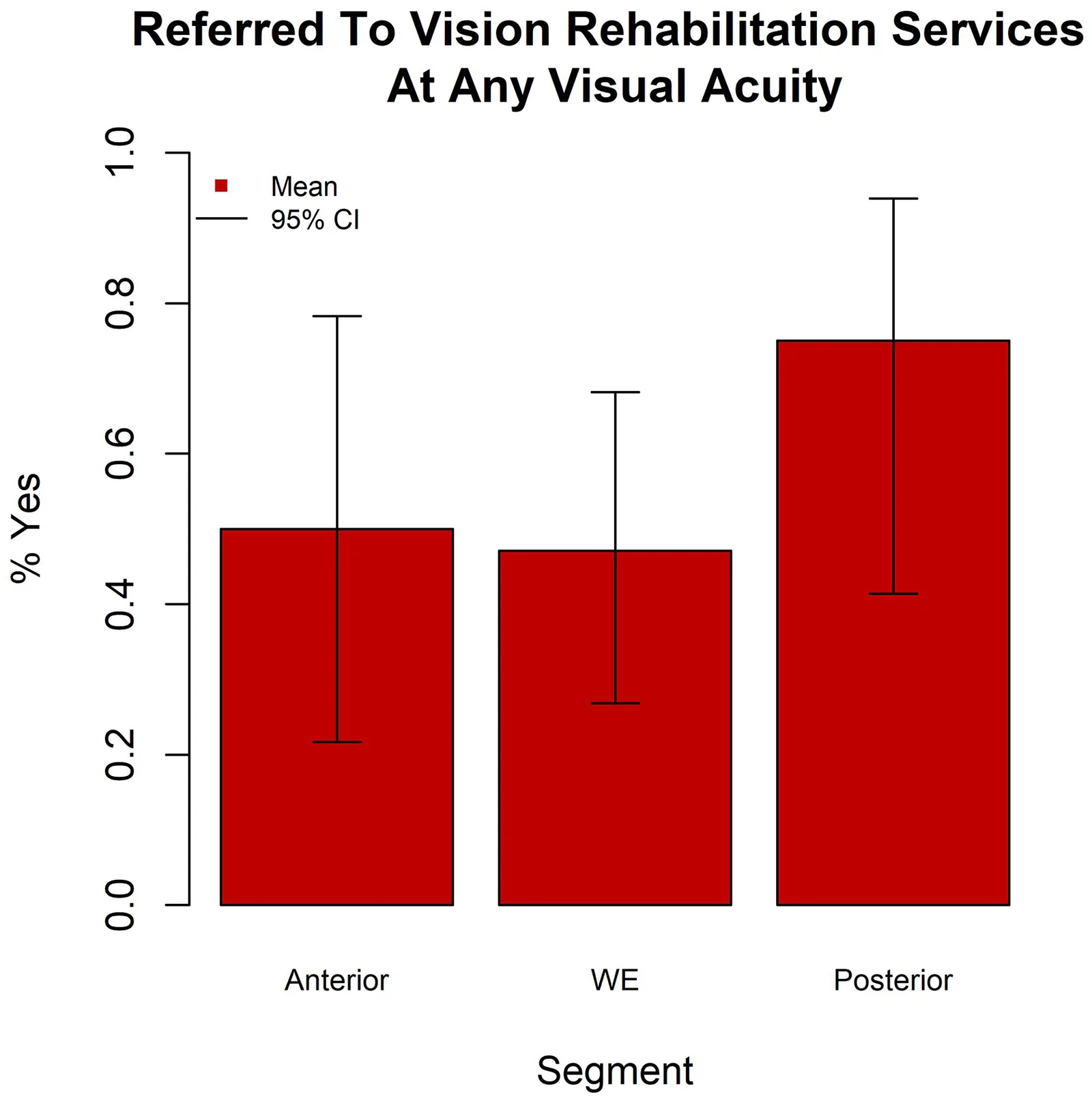
Refer to VRS at any visual acuity. Provider responses to referring patients to VRS at any visual acuity based on specialty. Bar heights display mean proportion with error bars as 95% confidence intervals.

Factors influencing referral decision-making to VRS were explored. When asked for a likely reason for the low referral rate, 52% of all respondents indicated that it is “very likely” that lack of patients asking about VRS is a main reason for low referrals. However, when asked about factors contributing to their decision-making for referring a patient to VRS, only 23% responded that patient initiating the discussion was “extremely important” in their decision to refer. When comparing specialties based on eye segment, patient-initiated discussion regarding VRS was more important for the decision-making for a referral from PS specialists (4.63 ± 0.22) as opposed to AS specialists (3.17 ± 0.65, p < 0.001) (Fig 3).

**Fig 3:**
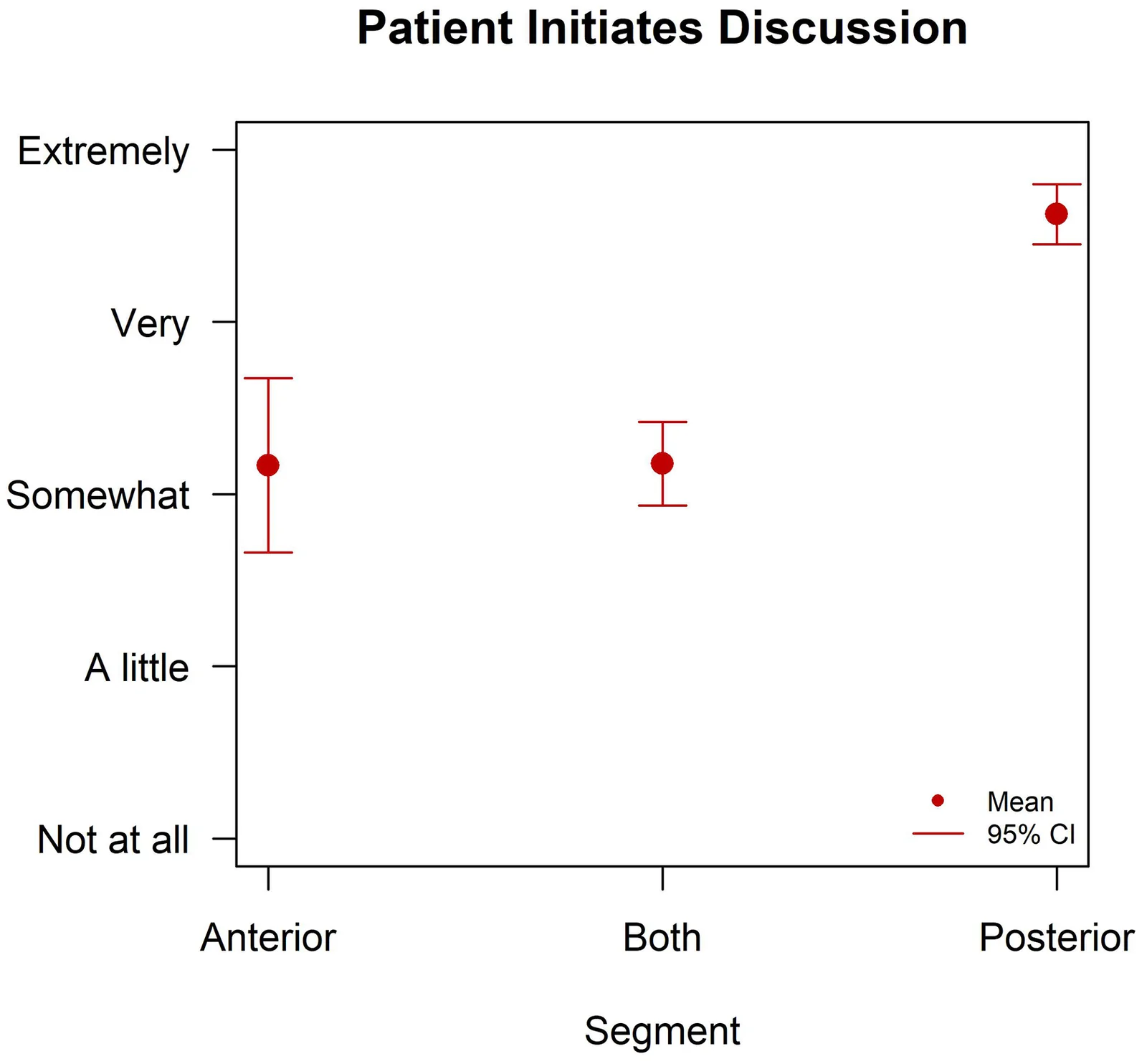
Patient initiates discussion. Patient-initiated discussion regarding VRS was important to the decision-making process to refer for PS specialists as opposed to AS specialists. Dots display mean Likert response with error bars as 95% confidence intervals.

When considering other factors, 42% of all respondents reported that the location of services in relation to where the patient lived as well as stage of the disease was “very important”. More PS specialists responded that location of services was important in their decision making (4.00 ± 0.33) in comparison to AS specialists who responded location had little impact (1.83 ± 0.48. p = 0.002) (Fig 4).

**Fig 4:**
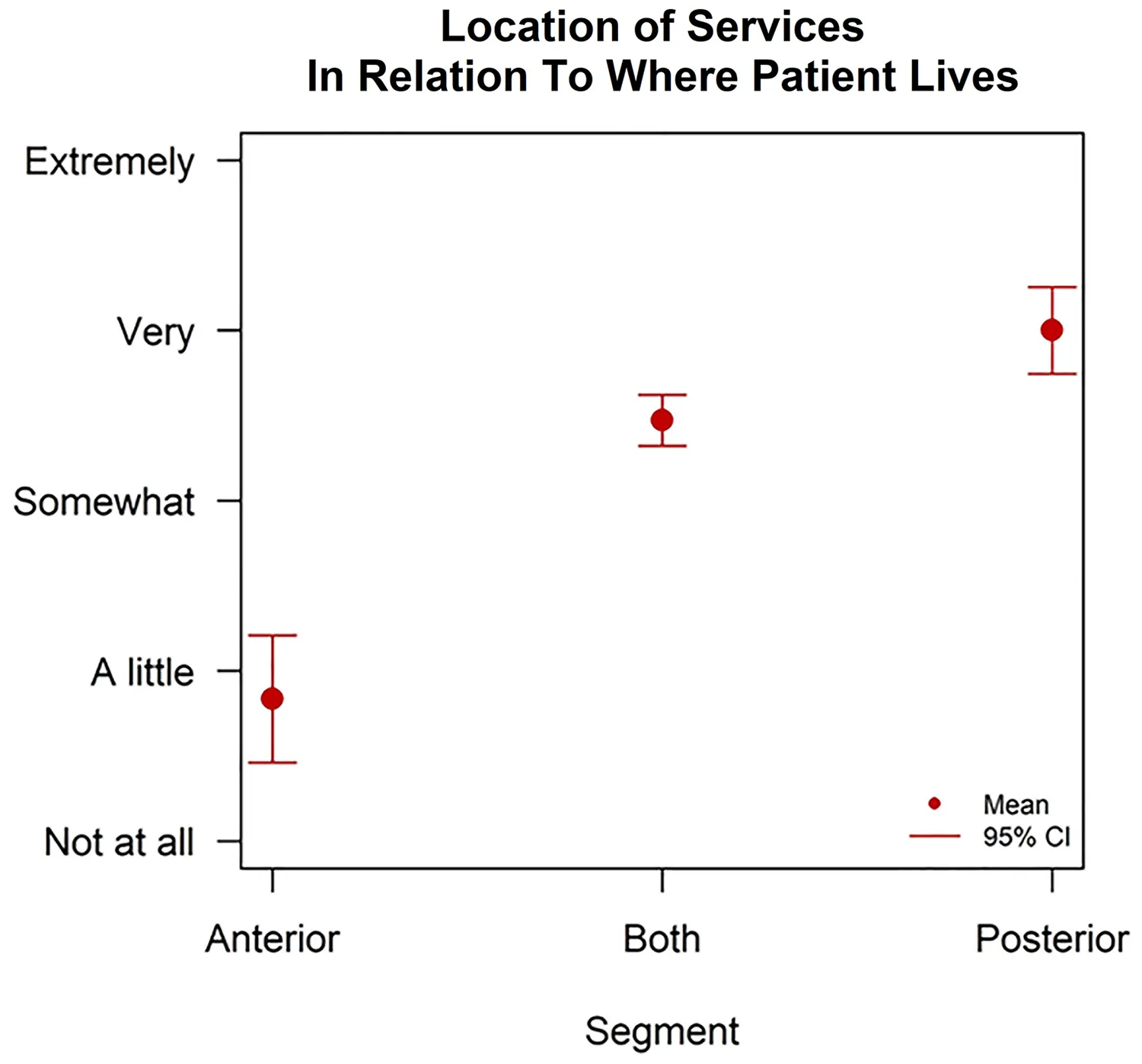
Location of service in relation to where the patient lives. Location of services in relation to where the patient lives was important to PS specialists in their decision making to refer patients to VRS whereas AS specialists responded that location had little impact. Dots display mean Likert response with error bars as 95% confidence intervals.

Regarding barriers for patients to receive VRS, 45% respondents indicated a lack of providers or long wait times were”quite a bit” of a barrier compared to cost of devices (29%), lack of transportation (29%) and the perception that the patient was not interested or would not go to the appointment (26%). This finding aligns with respondents’ perception on the difficulty of referring patients to VRS. Respondents felt the time it takes for the patient to be seen was “quite a bit” difficult (27%) in comparison to the time it takes to make a referral (7%) and the referral process (3%).

Over 35% of all respondents reported concerns about a patient’s driving ability as very important in their decision to refer. This concern was more impactful to PS specialists (4.00 ± 0.33) whereas AS specialists responded to this concern having little impact (2.83 ± 0.78, p = 0.012). Respondents with >20 YOP reported being more inclined to refer patients to VRS if patients-initiated discussion around driver’s license renewal or restrictions compared to respondents with <10 YOP (OR 21.9, CI 1.13–427.3) and respondents with 10–19 YOP (OR 79, CI 3.6–1741) (p < 0.05) (Fig 5). When asked if driver license restrictions were actively discussed with patients, 39% of all respondents reported it “extremely likely” and 36% of respondents reported it “somewhat likely”. When a patient is identified as unqualified to drive due to their vision impairment, only 26% of provider respondents reported it was “extremely likely” alternative transportation resources were discussed.

**Fig 5:**
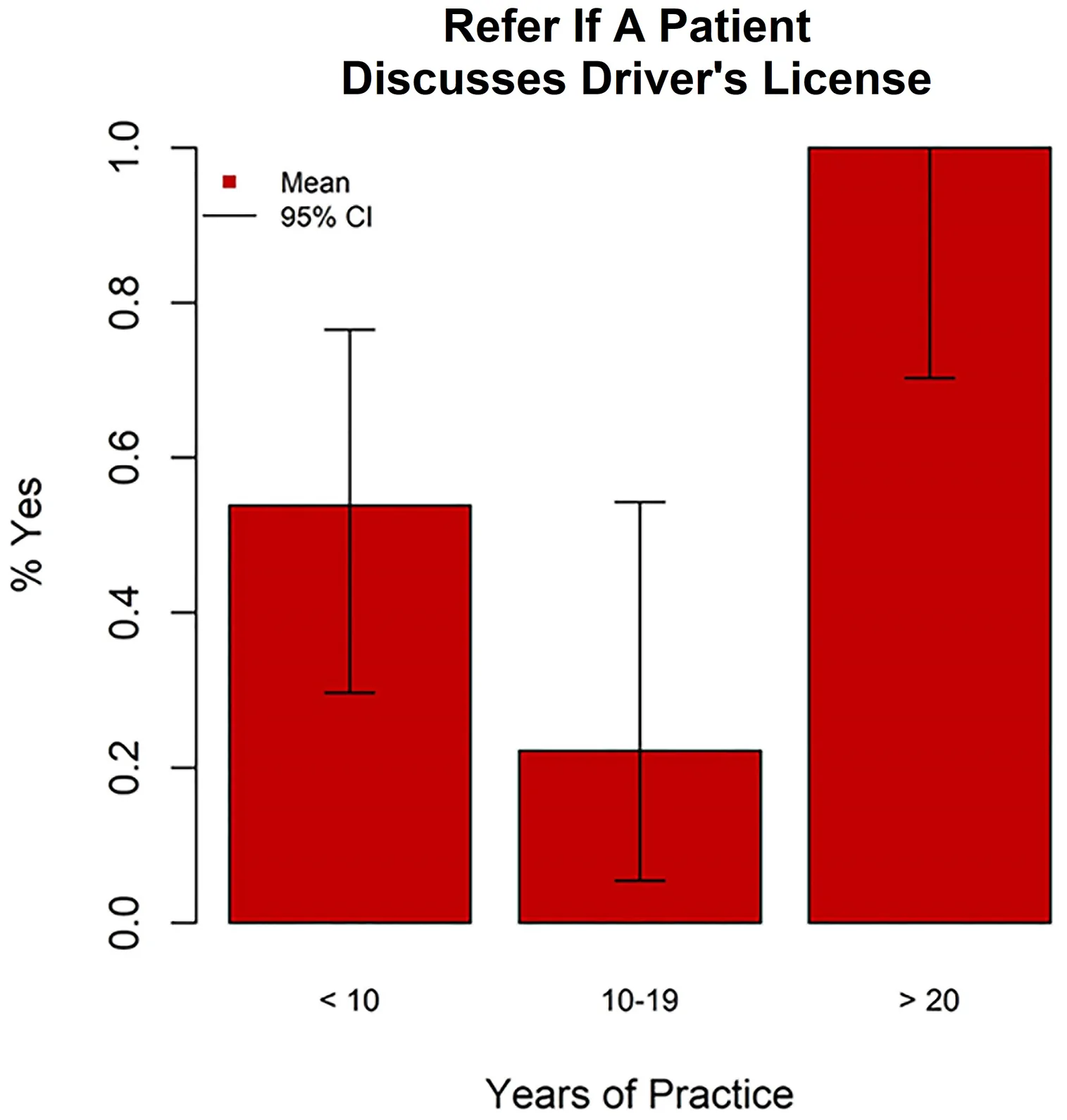
Refer if patient discusses driver’s license. Providers with > 20 YOP were more inclined to refer patients to VRS when patients initiated the discussion around driver’s license renewal or restrictions. Bar heights display mean proportion with error bars as 95% confidence intervals.

In addition to referring to in-clinic VRS, ECPs may refer patients to state or community organizations for VRS. Only 32% of respondents reported they were referring patients “very often” to community services such as nonprofit low vision organizations. Surprisingly, 42% of respondents reported “rarely” referring to state services. High volume practitioners reported referring more often to state services (2.87 ± 0.22) compared to low volume practitioners (2.00 ± 0.30) [33].

No difference was found between MDs and ODs on participation in continuing education courses about low vision or vision rehabilitation in the last two years. Interestingly, 75% (CI 32% − 96%) of PS specialists and 73% (CI 52% − 88%) of WE specialists reported participating in continuing education courses on the topic of low vision or vision rehabilitation in comparison to 0% (CI 0% − 3%) of AS specialists (OR 55.6 (CI 2.87–1077, p < 0.05) and OR 53.7 (CI 2.11–1374, p < 0.05) respectively) (Fig 6). Furthermore, 75% of female providers reported participating in continuing education courses on the same topic in the past 2 years versus 37% males (OR 4.38; 1.03, 18.68, p = 0.04).

**Fig 6:**
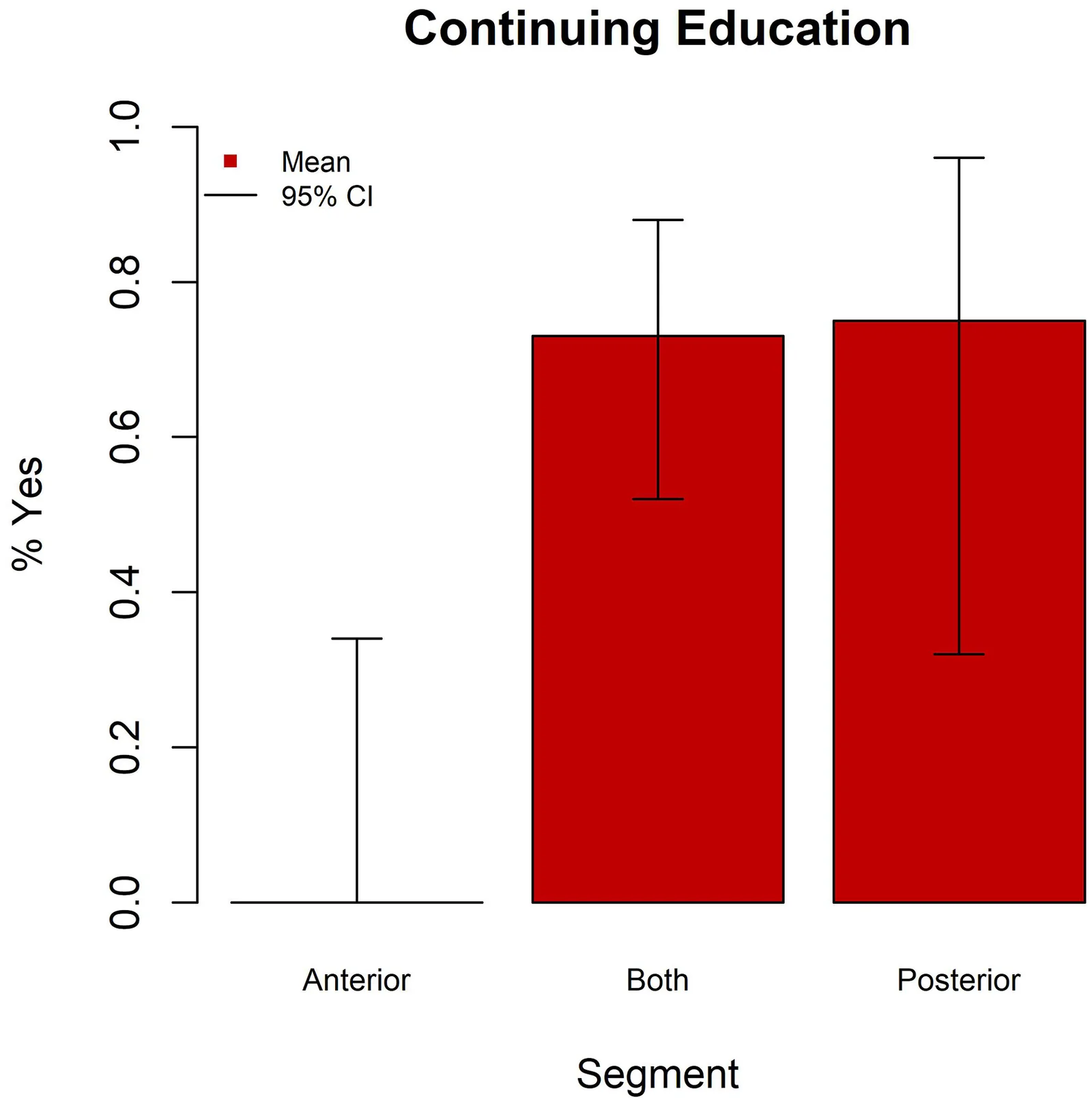
Continuing education. Seventy-five percent of PS specialists and 73.3% of WE specialists participated in continuing education on the topic of low vision/ vision rehabilitation in comparison to 0% of AS specialists. Bar heights display mean proportion with error bars as 95% confidence intervals.

## Discussion

Our results demonstrate that early career ECPs and PS specialists show greater awareness of the importance of initiating referrals to VRS regardless of patient visual acuity at our tertiary medical center. However, despite this awareness, over half of ECPs still attribute low referral rates to lack of patient inquiry. Most referrals to VRS from PS specialists and late career ECPs are triggered by patient inquiries regarding driving. Interestingly, while ECPs perceive lack of providers and long wait times as major barriers, our findings reveal overall, 61% of ECPs reported “never” to “sometimes” referring to community services. Thus, if majority of ECPs are not referring, their perception cannot be confirmed by their experience. Additionally, female ECPs, PS specialists, and WE specialists more frequently attended continuing education on low vision and vision rehabilitation topics. AS specialists may not attend continuing education on the same topics as many treatments and surgical outcomes utilized by these specialists greatly improve vision under their care [34,35]. However, it is important for them to be aware to refer patients when visual improvement does not occur.

### Provider perceptions and barriers to referral

Previous studies have consistently identified common factors affecting ECP referral decisions, including location of services, wait-times, availability, cost, patient inquiry about VRS, and driving considerations [24,36,37]. Our study confirms these patterns at our medical center persist despite the implementation of AAO PPP standard of care practices since 2017 [15]. Fifty-two percent of ECPs reported “very likely” and 23% reported “extremely likely” that a lack of VRS referrals were due to patients not inquiring about services. In terms of location of services to where the patient lives, 42% reported this as “very important” to their decision to refer to VRS. Regarding VRS availability and wait-times, 45% of ECPs reported this as “quite a bit” of a barrier for referring. Unfortunately, ECPs continue to wait for patients to initiate conversations about VRS before making referrals, most likely assuming they will receive negative reactions when broaching the topic [38,39]. Our results show 45% of ECPs assume it is “somewhat likely” patients will reject a referral to VRS. Fifty-two percent of ECPs assume a “somewhat” of a barrier to VRS is patient disinterest or lack of follow through with a referral while 26% felt this was “quite a bit” of a barrier.

ECPs demonstrate a higher awareness of AAO PPP standard of care guidelines, with our study showing about 77% of younger ECPs considering these guidelines when deciding on VRS referrals. Providers understand when to refer patients and recognize that VRS are both important and effective. However, our survey reveals a concerning disconnect: providers are making referral decisions based on their perceptions rather than established guidelines. For example, despite 75% PS specialists agreeing patients should be referred to VRS at any VA (Fig 2), many of these specialists report patient-initiated discussion regarding VRS was “very important” to “extremely important” in their VRS referral decision-making process (Fig 3). Fifty-two percent of respondents indicated that lack of patients inquiring about VRS mainly contributed as a reason for low referrals.

Despite guidelines recommending VRS at any visual acuity or stage of eye disease as standard of care, our study shows 26% of ECPs at our medical center still hesitate due to the perception that patients will resist referrals. This hesitation may stem from concerns about patients associating referrals with the stigma of being considered blind or doubts about the effectiveness of rehabilitation recommendations [32,34,40]. This perception is particularly problematic given research demonstrating that patients with mild to moderate vision impairment benefit more from VRS than those with severe vision impairment or blindness [41–43]. Health-related QOL becomes significantly impaired long before vision declines to severe levels [7,44]. Coker et al. found that more than 90% of patients with low vision had functional complaints due to their vision loss [45], while Shah et al. demonstrated that 100% of patients diagnosed with exudative age-related macular degeneration reported concerns about night driving, even with best corrected VA of 20/60 or better, with one-third developing depression [24]. The same study revealed that glaucoma patients are six times more likely to be involved in motor vehicle accidents.

### Addressing systemic barriers and misconceptions

A significant misconception among ECPs is that VRS represents an economic barrier for patients. In our study, 20% of respondents reported assuming cost of devices was a barrier to patients receiving VRS. However, treatment by ophthalmologists, optometrists or occupational therapists specializing in low vision and VRS is covered by Medicare and most health insurance carriers. While insurance plans typically do not cover device costs, numerous financial assistance programs exist throughout government and community low vision organizations [37]. This suggests ECPs lack awareness of available resources at our medical center, highlighting the need for further education in these areas.

Our study reveals a significant disconnect between perceived and actual barriers to VRS access. While 45% of ECPs perceive lack of sufficient VRS providers and long wait times as “quite a bit” of a barrier compared to transportation (29%), cost (29%), or perception about patient non-attendance (26%), only 32% refer “very often” to community services and 42% “rarely” refer to state services. This disparity suggests that ECPs, as gatekeepers of VRS, may contribute to the mismatch between potential need and actual VRS uptake at our medical center.

ECPs who participated in our survey demonstrate a pattern of selectivity when referring to VRS. Forty-two percent of ECPs reported location of services in relation to where the patient lives and stage of eye disease as a “very important” factor in their decision to refer to VRS. Studies have found that patients without health insurance are not referred due to cost assumptions, despite state services providing VRS at no cost [45]. Studies in the United States demonstrate that 36% of patients were not referred to VRS due to ECP-perceived barriers including poor health, declined cognitive function, aging, lack of social support, or projected low appointment attendance rates [44]. In our study 52% of ECPs reported low attendance as “somewhat” of a barrier to care whereas 26% felt this was “quite a bit” of a barrier. Importantly, the effects of vision impairment on QOL can both lead to these barriers and exacerbate them [1,25,33,46]. By being selective of VRS referrals, due to their own perceptions about access to care, ECPs may be contributing to the continued poor QOL outcomes associated with vision impairment.

### Patient-provider communication dynamics

Our results indicate that 52% of respondents view lack of patient awareness as the primary driver of low referral rates, a finding consistent with other studies showing that patient perspectives play an integral role in guiding referral decisions [32,34,47]. However, this perception is contradicted by research showing no increase in referrals from providers who claim to discuss VRS with patients [48]. This paradox suggests that ECPs continue to underrecognize the critical role of timely VRS referrals in optimizing vision-related functioning, addressing behavioral health, and maintaining independence and QOL.

The reality is that patient utilization of VRS is critically dependent on ECP recommendations and referrals, with clinician endorsement and advocacy playing essential roles in successful VRS delivery [14]. The current approach of waiting for patient-initiated conversations is problematic because patients unaware of VRS existence cannot initiate these discussions [14,40,49–51]. This dynamic may explain why still less than 15% of qualifying patients are referred to VRS despite involvement in guidelines incorporating VRS referrals as standard of care [21,35,45].

### Specialty-specific referral patterns

A notable finding from our study confirms a trend first observed by Keeffe et al. in 1996: PS specialists refer to VRS at significantly higher rates than other ECPs [52]. Over 25 years later, this pattern persists, with our study showing PS specialists (75%) are more cognizant of standard of care guidelines and attend continuing education on low vision and vision rehabilitation topics more frequently (75%) than AS specialists (0%) at our medical center.

Several factors could contribute to this disparity. PS specialists typically see higher patient volumes while actively providing certificate of legal blindness and discussing driving restrictions. Their more established patient base receiving VRS creates a positive feedback loop, triggering higher referral rates and more direct addressing of functional vision-related QOL concerns [52]. Additionally, PS specialists regularly perform retinal imaging and this tends to generate more VRS referrals [49].The prolonged patient relationships characteristic of PS specialists provides additional advantages. PS specialists see patients more frequently than AS and WE specialists, encounter more cases of irreversible vision loss, and follow patients for longer periods [53]. This extended relationship allows PS specialists to observe VA decline with degenerative eye disease progression and increases likelihood of receiving subjective complaints about visual function from patients.

In contrast, AS specialists may not sufficiently contribute to patient care longitudinally in order to consider details involving patient convenience with VRS or referral appropriateness [49]. For AS specialists focusing primarily on cataract surgery, patient visual ability for driving may not be a primary concern since most patients experience improved vision and visual function post-operatively, allowing them to return to driving unless other underlying diseases affect visual function. However, this perspective overlooks important opportunities. Patients with moderate to severe corneal disease, primarily seen by AS specialists, experience functional vision problems including impaired contrast sensitivity, color vision deficits, and glare sensitivity, and would greatly benefit from VRS to improve QOL. Since visual acuity is clinically measured with high contrast optotypes, these functional impacts often go unrecognized. Despite patients reporting reduced QOL due to eye disease, ECPs may reassure them that their vision is stable without referring to VRS to address QOL needs.

### Driving discussions and safety considerations

Driving emerges as a critical factor in VRS discussions, representing an essential component of daily living with major impacts on independence and serving as a primary motivator for patients VRS inquiries [46]. However, providers report uncertainty about when to address driving concerns or where to report driving difficulties. Most patients report that providers do not discuss whether they meet state vision requirements for driving licensure [45]. Our study shows a little over 35% of all respondents reported concerns about a patient’s driving ability as a very important decision to refer especially for PS specialists (50%). Only 39% of respondents reported actively discussing driving license restrictions with patients at our medical center.

This low percentage in respondents may result from ECPs’ unfamiliarity with state standards or recommended restrictions based on vision status. Many ECPs feel uncomfortable making active decisions about license restrictions or discussing driving cessation due to the significant impact on patients’ perception of independence and quality of life. Paradoxically, most people with vision impairment self-restrict their driving due to safety concerns from themselves, family or friends [54,55].

Given that driving is a necessity in many areas of the United States, particularly suburban and rural regions with limited public transportation, ECPs play a crucial role in helping patients make safe and informed decisions about driving status as vision worsens. The fear of having these discussions and potentially losing patient trust may influence decisions about VRS referrals.

### Future directions and recommendations

Our study points out several opportunities to improve VRS referral rates and access. Implementing continuing education opportunities that link categories of vision loss with specific types of visual function loss may help ECPs better understand VRS value for different patient categories and their impact on vision-related QOL domains [8]. Using current ICD-10 and ICD-11 codes alone with numerical definitions of low vision and blindness may be misleading as they fail to capture mild vision loss as well as other aspects of visual function, including contrast sensitivity, stereopsis, color vision, glare sensitivity, binocularity, and light/dark adaptation [56].

Establishing opportunities for enhanced provider-patient communication about vision loss could increase VRS access [30], though implementation remains challenging in high-volume practices, especially in tertiary care settings. Electronic health record-based clinical decision support systems represent a promising opportunity to increase referrals in tertiary care practices, as suggested by several studies [14,17,34]. Implementing referral alert tools in electronic health record systems has been specifically suggested as one method to overcome ECP barriers to referrals [14,34].

Implementing pre-visit surveys such as a modified visual function questionnaire [1] may boost provider confidence in broaching the topic of referring to VRS with their patient. The responses by the patient on the questionnaire would enable the provider to trigger a conversation about how VRS can help with QOL outcomes. This would reduce ECPs hesitancy about negative reaction or rejection to VRS referrals by the patient.

Ultimately, clinician endorsement and patient advocacy remain essential for improving VRS delivery [14]. As tertiary medical centers serve the greatest number of visually impaired people outside of government agencies and rehabilitation centers [31], ECPs in these settings play particularly critical roles as either facilitators or barriers to VRS access [13]. Despite onsite VRS availability, our study confirms that lack of timely referrals persists at our medical center, emphasizing the need for systematic approaches to improve referral practices and ultimately enhance patient outcomes and QOL.

### Limitations

This study has some limitations. It was conducted at a single tertiary academic medical center, reflecting an intentional focus on a pre-defined target population. As is typical in finite-population survey designs, the results are not generalizable beyond this setting, which should be viewed as a characteristic of the study design rather than a methodological shortcoming. Although some sub-specialty groups had low sample sizes, the application of the finite population correction yields valid inferences and unbiased estimates for the sampled population. Also, response and social desirability bias to the survey questions may have occurred where the expected answer is provided in lieu of an accurate and authentic answer. Expanding the study to various clinical settings would yield more robust information about current perceptions by ECP in various practice settings. A complementary study of patient awareness and attitudes to low vision services would further validate and contextualize the findings reported here.

## Conclusion

Our study shows younger providers and PS specialists are aware of standard of care guidance on timely referrals to VRS at our medical center. However, referral rates continue to be low due to provider perception and biases affecting who qualifies for services, availability of services, location of services relative to the patient, comfort level of discussing services and who will keep appointment for services. ECPs are the gatekeepers to VRS due to their training as well as implementations of VRS as standard of care. Providers need to understand they must refer based on a patient’s functional vision complaint as well as standard of care practices. Level of VA loss alone or waiting for patient inquiry cannot be solely relied on for best practices. Active provider-patient conversations addressing vision loss early on with eye disease progression and timely referrals to VRS are important for sustaining patient’s QOL domains, independence and psychosocial wellbeing. Specialty centric continuing education courses for ECPs may be helpful for providers to understand how the diseases they treat affect visual function and QOL. Implementing clinic decision support systems may also help remind providers when a discussion about VRS is appropriate with patients.

## Supporting information

S1 File Appendix. Eye care provider survey regarding the visually impaired population.
(DOCX)
