# Supplementary material for: Understanding decision making factors influencing referrals to vision rehabilitation services at a tertiary medical center
Source: PLoS One. 2026 Oct 26;21(10):e0359154. doi: 10.1371/journal.pone.0359154 (PMC13704866; doi:10.1371/journal.pone.0359154)
Supplement: Supplementary file 1 [file pone.0359154.s001.docx]

**S1 Appendix:** **Eye care provider survey regarding the visually impaired population.**

**Thank you for your interest in our study.**

**Our goal is to understand provider perception of vision impairment and vision rehabilitation services. Your responses will enable us to recognize and address gaps to improve the process of vision rehabilitation and referrals for the overall psychosocial wellbeing of patients.**

**We invite you to complete this online questionnaire, which should take about 10 minutes to complete. All of your responses to this questionnaire are confidential. Any information you share will only be reported in group form and will not be reported in a way that would allow you to be personally identified.**

**Instructions to complete the questionnaire:**

**To choose a response, click on the button that corresponds to your answer. If you would like to change your answer, click on a different button. You may go back to earlier screens by clicking the 'BACK' button at the bottom of the screen. To advance to the next screen click the 'NEXT' button.**

**If you start the questionnaire and are unable to finish it, you can exit by simply closing the browser window and return to it later by clicking again on the link in your email. This will take you back to the next unanswered question. If there is a question you leave blank, you will be informed that it was left blank. You may then either return to answer that question or continue on to the next question. You are not required to answer any question you would prefer to skip.**

**After the entire questionnaire has been completed and all the data are ready to be submitted, please click on the 'SUBMIT' button on the last screen. Once you click 'SUBMIT' you will not be able to re-enter the questionnaire.**

**We appreciate your time and effort in assisting us by completing this web-based questionnaire.**

**Please click next to begin the questionnaire now.**

|  | **01.** |  | **The first questions are about you and your practice.**  **Are you an optometrist or ophthalmologist?** | |
| --- | --- | --- | --- | --- |
|  |  |  |  |  |
|  |  |  |  | Optometrist |
|  |  |  |  | Ophthalmologist |
|  |  |  |  |  |

|  | **02.** |  | **Which part of the eye do you specialize in?** | |
| --- | --- | --- | --- | --- |
|  |  |  |  |  |
|  |  |  |  | Anterior segment |
|  |  |  |  | Posterior segment |
|  |  |  |  | Both anterior and posterior segments |
|  |  |  |  |  |

|  | **03.** |  | **For how many years have you been in practice?** | |
| --- | --- | --- | --- | --- |
|  |  |  |  |  |
|  |  |  |  | Less than 10 years |
|  |  |  |  | 10 to 19 years |
|  |  |  |  | 20 to 29 years |
|  |  |  |  | 30 years or more |
|  |  |  |  |  |

|  | **04.** |  | **At how many locations do you currently practice?** | |
| --- | --- | --- | --- | --- |
|  |  |  |  |  |
|  |  |  |  | One |
|  |  |  |  | Two |
|  |  |  |  | Three |
|  |  |  |  | More than three |
|  |  |  |  |  |

|  | **05.** |  | **Including all the locations where you practice, about how many patients do you see in an average day?** | |
| --- | --- | --- | --- | --- |
|  |  |  |  |  |
|  |  |  |  | Less than 10 |
|  |  |  |  | 10 to 19 |
|  |  |  |  | 20 to 29 |
|  |  |  |  | 30 to 39 |
|  |  |  |  | 40 or more |
|  |  |  |  |  |

|  | **06.** |  | **Next, we have some questions about when a patient should be referred for low vision services.**  **At what visual acuity (Snellen) should a patient be referred for low vision services?** | |
| --- | --- | --- | --- | --- |
|  |  |  |  |  |
|  |  |  |  | Any visual acuity |
|  |  |  |  | 20/50 to 20/60 |
|  |  |  |  | 20/70 to 20/150 |
|  |  |  |  | 20/200 to 20/400 |
|  |  |  |  | worse than 20/400 |
|  |  |  |  |  |

|  | **07.** | |  | **In terms of vision field loss, when would you refer a patient for low vision services in each of the following situations?**  **Would you refer a patient for low vision services if…** | | | | | | | |  |
| --- | --- | --- | --- | --- | --- | --- | --- | --- | --- | --- | --- | --- |
|  |  | |  |  |  |  | | |  | | |  |
|  |  | |  |  |  | **Yes** | | | **No** | | |  |
|  |  |  |  | **a.** | …their visual field limitation is greater than 20 degrees central field of vision in the better seeing eye? |  |  |  |  |  |  |  |
|  |  |  |  | **b.** | …their visual field limitation is less than or equal to 20 degrees central field of vision in the better seeing eye? |  |  |  |  |  |  |  |
|  |  |  |  | **c.** | …they self-report impact on activities of daily living or mobility due to visual field loss? |  |  |  |  |  |  |  |
|  |  |  |  | **d.** | …they bring up renewing their driver’s license? |  |  |  |  |  |  |  |
|  |  | |  |  |  |  | | |  | | |  |

|  | **08.** | |  | **What would you consider if a patient is complaining about their vision, but their visual acuity is better than 20/40 according to the Snellen Acuity chart?**  **Would you consider…** | | | | | | | |  |
| --- | --- | --- | --- | --- | --- | --- | --- | --- | --- | --- | --- | --- |
|  |  | |  |  |  |  | | |  | | |  |
|  |  | |  |  |  | **Yes** | | | **No** | | |  |
|  |  |  |  | **a.** | …contrast sensitivity? |  |  |  |  |  |  |  |
|  |  |  |  | **b.** | …glare sensitivity? |  |  |  |  |  |  |  |
|  |  |  |  | **c.** | …central or para central scotomas? |  |  |  |  |  |  |  |
|  |  |  |  | **d.** | …design of glasses, that is progressive additional lenses vs. bifocals vs. trifocals? |  |  |  |  |  |  |  |
|  |  | |  |  |  |  | | |  | | |  |

|  | **09.** | |  | **Even though our clinics offer low vision services, only about 10% of patients with moderate vision loss, that is 20/70 through 20/200, are referred to them.**  **How likely is each of the following a reason for the small number of referrals to low vision services?** | | | | | | | | | | | | | | | | |  |
| --- | --- | --- | --- | --- | --- | --- | --- | --- | --- | --- | --- | --- | --- | --- | --- | --- | --- | --- | --- | --- | --- |
|  |  | |  |  |  |  | | |  | | |  | | |  | | |  | | |  |
|  |  | |  |  |  | **Not at all likely** | | | **A little likely** | | | **Somewhat likely** | | | **Very likely** | | | **Extremely likely** | | |  |
|  |  |  |  | **a.** | Low vision services have not been discussed with patients |  |  |  |  |  |  |  |  |  |  |  |  |  |  |  |  |
|  |  |  |  | **b.** | Patients do not ask about low vision services |  |  |  |  |  |  |  |  |  |  |  |  |  |  |  |  |
|  |  |  |  | **c.** | Patients will reject low vision services |  |  |  |  |  |  |  |  |  |  |  |  |  |  |  |  |
|  |  |  |  | **d.** | Low vision services are negatively characterized by patients |  |  |  |  |  |  |  |  |  |  |  |  |  |  |  |  |
|  |  |  |  | **e.** | Low vision services will not help |  |  |  |  |  |  |  |  |  |  |  |  |  |  |  |  |
|  |  |  |  | **f.** | Most doctors prefer to wait until no further treatment helps before referring |  |  |  |  |  |  |  |  |  |  |  |  |  |  |  |  |
|  |  |  |  | **g.** | Often doctors do not find out the outcome for services they refer patients to |  |  |  |  |  |  |  |  |  |  |  |  |  |  |  |  |
|  |  | |  |  |  |  | | |  | | |  | | |  | | |  | | |  |

|  | **10.** | |  | **How important is each of the following in your decision to refer patients to low vision services?** | | | | | | | | | | | | | | | | |  |
| --- | --- | --- | --- | --- | --- | --- | --- | --- | --- | --- | --- | --- | --- | --- | --- | --- | --- | --- | --- | --- | --- |
|  |  | |  |  |  |  | | |  | | |  | | |  | | |  | | |  |
|  |  | |  |  |  | **Not at all** | | | **A little** | | | **Somewhat** | | | **Very** | | | **Extremely** | | |  |
|  |  |  |  | **a.** | Location of services in relation to where you practice |  |  |  |  |  |  |  |  |  |  |  |  |  |  |  |  |
|  |  |  |  | **b.** | Location of services in relation to where the patient lives |  |  |  |  |  |  |  |  |  |  |  |  |  |  |  |  |
|  |  |  |  | **c.** | Negative patient interaction |  |  |  |  |  |  |  |  |  |  |  |  |  |  |  |  |
|  |  |  |  | **d.** | Eye condition |  |  |  |  |  |  |  |  |  |  |  |  |  |  |  |  |
|  |  |  |  | **e.** | Stage of eye disease |  |  |  |  |  |  |  |  |  |  |  |  |  |  |  |  |
|  |  |  |  | **f.** | Concerns about patient’s driving ability |  |  |  |  |  |  |  |  |  |  |  |  |  |  |  |  |
|  |  |  |  | **g.** | Patient initiates the discussion |  |  |  |  |  |  |  |  |  |  |  |  |  |  |  |  |
|  |  | |  |  |  |  | | |  | | |  | | |  | | |  | | |  |

|  | **11.** | |  | **How much of a barrier is each of the following to patients seeking low vision services?** | | | | | | | | | | | | | | | | |  |
| --- | --- | --- | --- | --- | --- | --- | --- | --- | --- | --- | --- | --- | --- | --- | --- | --- | --- | --- | --- | --- | --- |
|  |  | |  |  |  |  | | |  | | |  | | |  | | |  | | |  |
|  |  | |  |  |  | **Not at all** | | | **A little** | | | **Somewhat** | | | **Quite a bit** | | | **A great deal** | | |  |
|  |  |  |  | **a.** | Cost of devices |  |  |  |  |  |  |  |  |  |  |  |  |  |  |  |  |
|  |  |  |  | **b.** | Patients not interested or would not go |  |  |  |  |  |  |  |  |  |  |  |  |  |  |  |  |
|  |  |  |  | **c.** | Lack of enough providers or long wait times to receive services |  |  |  |  |  |  |  |  |  |  |  |  |  |  |  |  |
|  |  |  |  | **d.** | Patient’s perception towards services |  |  |  |  |  |  |  |  |  |  |  |  |  |  |  |  |
|  |  |  |  | **e.** | Difficult referral process |  |  |  |  |  |  |  |  |  |  |  |  |  |  |  |  |
|  |  |  |  | **f.** | Patients’ lack of transportation to visits |  |  |  |  |  |  |  |  |  |  |  |  |  |  |  |  |
|  |  | |  |  |  |  | | |  | | |  | | |  | | |  | | |  |

|  | **12.** | |  | **How much of a difficulty is each of the following when referring patients to vision rehabilitation services?** | | | | | | | | | | | | | | | | |  |
| --- | --- | --- | --- | --- | --- | --- | --- | --- | --- | --- | --- | --- | --- | --- | --- | --- | --- | --- | --- | --- | --- |
|  |  | |  |  |  |  | | |  | | |  | | |  | | |  | | |  |
|  |  | |  |  |  | **Not at all** | | | **A little** | | | **Somewhat** | | | **Quite a bit** | | | **A great deal** | | |  |
|  |  |  |  | **a.** | Process of referral |  |  |  |  |  |  |  |  |  |  |  |  |  |  |  |  |
|  |  |  |  | **b.** | Time it takes to make a referral |  |  |  |  |  |  |  |  |  |  |  |  |  |  |  |  |
|  |  |  |  | **c.** | Time it takes for the patient to be seen |  |  |  |  |  |  |  |  |  |  |  |  |  |  |  |  |
|  |  | |  |  |  |  | | |  | | |  | | |  | | |  | | |  |

|  | **13.** | |  | **In the last year, how often have you referred patients to…** | | | | | | | | | | | | | | | | |  |
| --- | --- | --- | --- | --- | --- | --- | --- | --- | --- | --- | --- | --- | --- | --- | --- | --- | --- | --- | --- | --- | --- |
|  |  | |  |  |  |  | | |  | | |  | | |  | | |  | | |  |
|  |  | |  |  |  | **Never** | | | **Rarely** | | | **Sometimes** | | | **Very**  **often** | | | **Extremely**  **often** | | |  |
|  |  |  |  | **a.** | …community low vision services such as Wisconsin Council of the Blind and Visually Impaired, Vision Forward, etcetera? |  |  |  |  |  |  |  |  |  |  |  |  |  |  |  |  |
|  |  |  |  | **b.** | …state low vision services such as Office for the Blind and Visually Impaired, Wisconsin Center for the Blind and Visually Impaired, Department of Vocational Rehabilitation, etcetera? |  |  |  |  |  |  |  |  |  |  |  |  |  |  |  |  |
|  |  | |  |  |  |  | | |  | | |  | | |  | | |  | | |  |

|  | **14.** |  | **If you feel a patient’s vision is affecting their behavioral or mental health, do you address this issue with them?** | |
| --- | --- | --- | --- | --- |
|  |  |  |  |  |
|  |  |  |  | Yes |
|  |  |  |  | No |
|  |  |  |  |  |

**[Programmer Note: display only if YES at previous question]**

|  | **15.** | |  | **In the last year, when a patient’s vision has affected their behavioral or mental health, how often have you…** | | | | | | | | | | | | | | | | |  |
| --- | --- | --- | --- | --- | --- | --- | --- | --- | --- | --- | --- | --- | --- | --- | --- | --- | --- | --- | --- | --- | --- |
|  |  | |  |  |  |  | | |  | | |  | | |  | | |  | | |  |
|  |  | |  |  |  | **Never** | | | **Rarely** | | | **Sometimes** | | | **Very**  **often** | | | **Extremely**  **often** | | |  |
|  |  |  |  | **a.** | …sent a note to their Primary Care Provider? |  |  |  |  |  |  |  |  |  |  |  |  |  |  |  |  |
|  |  |  |  | **b.** | …shared resources with them? |  |  |  |  |  |  |  |  |  |  |  |  |  |  |  |  |
|  |  |  |  | **c.** | …put in a referral to behavioral health? |  |  |  |  |  |  |  |  |  |  |  |  |  |  |  |  |
|  |  |  |  | **d.** | …put in a referral to social work services? |  |  |  |  |  |  |  |  |  |  |  |  |  |  |  |  |
|  |  | |  |  |  |  | | |  | | |  | | |  | | |  | | |  |

|  | **16.** |  | **When you see a patient struggling to get around in the clinic, how likely are you to address orientation and mobility with them?** | |
| --- | --- | --- | --- | --- |
|  |  |  |  |  |
|  |  |  |  | Not at all |
|  |  |  |  | A little |
|  |  |  |  | Somewhat |
|  |  |  |  | Very |
|  |  |  |  | Extremely |
|  |  |  |  |  |

|  | **17.** | |  | **In the last year, when discussing difficult or distressing information with a patient, how often have you…** | | | | | | | | | | | | | | | | |  |
| --- | --- | --- | --- | --- | --- | --- | --- | --- | --- | --- | --- | --- | --- | --- | --- | --- | --- | --- | --- | --- | --- |
|  |  | |  |  |  |  | | |  | | |  | | |  | | |  | | |  |
|  |  | |  |  |  | **Never** | | | **Rarely** | | | **Sometimes** | | | **Very**  **often** | | | **Extremely**  **often** | | |  |
|  |  |  |  | **a.** | …gone over images or results? |  |  |  |  |  |  |  |  |  |  |  |  |  |  |  |  |
|  |  |  |  | **b.** | …discussed disease process? |  |  |  |  |  |  |  |  |  |  |  |  |  |  |  |  |
|  |  |  |  | **c.** | …discussed how the disease progression will affect vision over time? |  |  |  |  |  |  |  |  |  |  |  |  |  |  |  |  |
|  |  |  |  | **d.** | …discussed the effects on activities of daily living, vocation or driving? |  |  |  |  |  |  |  |  |  |  |  |  |  |  |  |  |
|  |  |  |  | **e.** | …left time for questions? |  |  |  |  |  |  |  |  |  |  |  |  |  |  |  |  |
|  |  |  |  | **f.** | …provided resources? |  |  |  |  |  |  |  |  |  |  |  |  |  |  |  |  |
|  |  |  |  | **g.** | …provided an action plan? |  |  |  |  |  |  |  |  |  |  |  |  |  |  |  |  |
|  |  | |  |  |  |  | | |  | | |  | | |  | | |  | | |  |

|  | **18.** |  | **What do you base a patient’s status of legal blindness on?** | |
| --- | --- | --- | --- | --- |
|  |  |  |  |  |
|  |  |  |  | Visual acuity only |
|  |  |  |  | Visual field only |
|  |  |  |  | Both visual acuity and visual field |
|  |  |  |  |  |

|  | **19.** |  | **For this question, please consider the US standard for legal blindness as defined by the Social Security Administration:**   - **Part 1: A visual acuity of 20/200 or worse in the better seeing eye with best conventional correction** - **Part 2: A visual field (the total area an individual can see without moving the eyes from side to side) of 20 degrees or less (also called tunnel vision) in the better seeing eye.**   **How likely are you to actively address legal blindness with patients who meet the standard?** | |
| --- | --- | --- | --- | --- |
|  |  |  |  |  |
|  |  |  |  | Not at all likely |
|  |  |  |  | A little likely |
|  |  |  |  | Somewhat likely |
|  |  |  |  | Very likely |
|  |  |  |  | Extremely likely |
|  |  |  |  |  |

**[Programmer note: Display only if answer is other than “not at all likely” at the previous question]**

|  | **20.** |  | **In the last year, when you have actively addressed legal blindness with a patient, how often have you provided a certificate of legal blindness?** | |
| --- | --- | --- | --- | --- |
|  |  |  |  |  |
|  |  |  |  | Never |
|  |  |  |  | Rarely |
|  |  |  |  | Sometimes |
|  |  |  |  | Very often |
|  |  |  |  | Always |
|  |  |  |  |  |

|  | **21.** |  | **The State of Wisconsin Department of Transportation vision standards for an unrestricted driver’s license is: Best corrected visual acuity of 20/40 or better in each eye and 70 degrees field of vision from the center in each eye.**  **When a patient’s visual acuity or visual field does not meet the criteria for an unrestricted driver’s license, how likely are you to actively discuss license restrictions with them?** | |
| --- | --- | --- | --- | --- |
|  |  |  |  |  |
|  |  |  |  | Not at all likely |
|  |  |  |  | A little likely |
|  |  |  |  | Somewhat likely |
|  |  |  |  | Very likely |
|  |  |  |  | Extremely likely |
|  |  |  |  |  |

|  | **22.** |  | **When you are aware a patient cannot or should not drive, how likely are you to actively discuss transportation resources with them?** | |
| --- | --- | --- | --- | --- |
|  |  |  |  |  |
|  |  |  |  | Not at all likely |
|  |  |  |  | A little likely |
|  |  |  |  | Somewhat likely |
|  |  |  |  | Very likely |
|  |  |  |  | Extremely likely |
|  |  |  |  |  |

|  | **23.** |  | **Overall, how well do your patients understand their eye condition?** | |
| --- | --- | --- | --- | --- |
|  |  |  |  |  |
|  |  |  |  | Not at all well |
|  |  |  |  | A little well |
|  |  |  |  | Somewhat well |
|  |  |  |  | Very well |
|  |  |  |  | Extremely well |
|  |  |  |  |  |

**[Programmer note: Display only if answer at the previous question is other than “very well” or “extremely well”]**

|  | **24.** | |  | **If a patient has little to no understanding of their eye condition and its impact on visual function, how helpful would each of the following be?** | | | | | | | | | | | | | | | | |  |
| --- | --- | --- | --- | --- | --- | --- | --- | --- | --- | --- | --- | --- | --- | --- | --- | --- | --- | --- | --- | --- | --- |
|  |  | |  |  |  |  | | |  | | |  | | |  | | |  | | |  |
|  |  | |  |  |  | **Not at all**  **helpful** | | | **A little**  **helpful** | | | **Somewhat helpful** | | | **Very**  **helpful** | | | **Extremely**  **helpful** | | |  |
|  |  |  |  | **a.** | Brochures or online resources |  |  |  |  |  |  |  |  |  |  |  |  |  |  |  |  |
|  |  |  |  | **b.** | Counseling |  |  |  |  |  |  |  |  |  |  |  |  |  |  |  |  |
|  |  |  |  | **c.** | A patient care advocate |  |  |  |  |  |  |  |  |  |  |  |  |  |  |  |  |
|  |  |  |  | **d.** | Social work services |  |  |  |  |  |  |  |  |  |  |  |  |  |  |  |  |
|  |  |  |  | **e.** | Support groups |  |  |  |  |  |  |  |  |  |  |  |  |  |  |  |  |
|  |  | |  |  |  |  | | |  | | |  | | |  | | |  | | |  |

|  | **25.** |  | **It is helpful for us to know which services our staff do and do not know about.**  **Social workers are available to help families cope with a new diagnosis of vision loss. We have had a department-assigned social worker since 2017.**  **In the last year, how often have you referred patients to the department-assigned social worker?** | |
| --- | --- | --- | --- | --- |
|  |  |  |  |  |
|  |  |  |  | Never |
|  |  |  |  | Rarely |
|  |  |  |  | Sometimes |
|  |  |  |  | Very often |
|  |  |  |  | Always |
|  |  |  |  |  |

| **26.** |  | **Finally, we have some questions about you and your education.**  **What is your gender identity?** | |
| --- | --- | --- | --- |
|  |  |  |  |
|  |  |  | Man |
|  |  |  | Woman |
|  |  |  | Nonbinary |
|  |  |  | Not listed, please tell us: |
|  |  |  |  |

|  | **27.** | |  | **Have you ever received formal education or training on…** | | | | | | | |  |
| --- | --- | --- | --- | --- | --- | --- | --- | --- | --- | --- | --- | --- |
|  |  | |  |  |  |  | | |  | | |  |
|  |  | |  |  |  | **Yes** | | | **No** | | |  |
|  |  |  |  | **a.** | …how to discuss difficult or distressing information with your patients? |  |  |  |  |  |  |  |
|  |  |  |  | **b.** | …low vision or vision rehabilitation? |  |  |  |  |  |  |  |
|  |  | |  |  |  |  | | |  | | |  |

**[Programmer note: Display only if YES at “low vision or vision rehabilitation?” in the previous question**

|  | **28** |  | **In the past 2 years, have you participated in any continuing education about low vision or vision rehabilitation?** | |
| --- | --- | --- | --- | --- |
|  |  |  |  |  |
|  |  |  |  | Yes |
|  |  |  |  | No |
|  |  |  |  |  |
