# Supplementary material for: Understanding decision making factors influencing referrals to vision rehabilitation services at a tertiary medical center
Source: PLoS One. 2026 Oct 26;21(10):e0359154. doi: 10.1371/journal.pone.0359154 (PMC13704866; doi:10.1371/journal.pone.0359154)
Supplement: Supplementary file 2 [file pone.0359154.s003.docx]

February 12, 2026.

**Subject: Revision Submission – Manuscript PONE-D-25-43062**

**Dear Dr. Martinez,**

Thank you for the opportunity to submit our revised manuscript “Understanding decision making factors influencing referrals to vision rehabilitation services at a tertiary medical center” for consideration in PLOS ONE. We would like to extend our appreciation for the input and suggestions provided by both you and the reviewers. We have carefully considered all comments, and the revisions have significantly improved our manuscript. We have provided an explanation of changes below. All line references refer to the unmarked revised “Manuscript.”

**Response to Editor:**

We have made all revisions to ensure that they align with the journal requirements and have now provided the following statement regarding the funder’s role “The funders had no role in study design, data collection and analysis, decision to publish, or preparation of the manuscript” in the financial disclosure and in the cover letter as requested. A de-identified data set is now shared. As requested, a full ethics statement has been added in the ‘Methods’ section in the manuscript file starting from line 98. In addition, although a laboratory protocol per se does not exist for this survey-based study, a copy of the survey was shared as Appendix 1. To honor the reviewers’ feedback regarding acronym use throughout the manuscript, we have condensed some of the less commonly used acronyms and have edited the titles and legends for Figures 1, 2, 4 and 5 to reflect these changes.

**Reviewer #1:**

1) **“…there are several grammatical errors in the body of the text. The authors need to better define their acronyms as some are used without definition…”**

Response: Thank you for taking the time to review our manuscript and for the detailed feedback. We have revised and corrected grammatical errors throughout the body of the text. As explained below, we have also modified the acronyms in the manuscript to facilitate flow and understanding.

All acronyms were revised and removed from the abstract. The acronyms VI, CE, ADL and VA were removed from the entire manuscript and only acronyms used most frequently remain in body of the text and are now defined including AAO PPP (line 49).

The specific sections provided in your review with grammatical errors have been revised and corrected. Line 68 has been revised and is now line 75. Line 100 has been revised and is now line 109. Line 122 has been revised and is now line 142.

2) **“I would have liked to have seen a table describing the demographic profile of the respondents (as described in lines102-105) and then broken up by their survey responses.”**

Response: Thank you for the suggestion. A table (see Table 1) and a description of the demographic profile of respondents have been added to the beginning of the results section (lines 132 to 137). In addition, differences in responses between genders were not found to be statistically significant for all results except for continuing education. This has been now added to the manuscript. Demographic profile is now added in the conclusion section.

3) **You also state that "The psychosocial impact will be assessed in a separate report", however, I think your paper would benefit greatly if you discussed the psychosocial impact here in this paper together with your survey results".**

Response: Thank you for your interest in the perceived patient psychosocial impact by providers. Psychosocial impact is an under reported issue that we believe will be best addressed in a separate report. We believe that sufficient data has been collected to justify a focused manuscript on this topic alone, rather than including it in our report on provider bias in referrals, where the findings could be diluted.

4) **You state that "Consent was not required by the Institutional Review Board as the study was not considered to involve human subjects." The reviewer would like further clarification.**

Response: Please refer to the IRB approval letter under ‘Others’ in the submission. This study went through full IRB review and was deemed as not research involving human subjects as defined by the Department of Health and Human Services and Food and Drug Administration regulations (Reference UW IRB ID# 2023-0119). Thus, obtaining consent for participation was not required. The letter does not provide any further information about the determination.

5) **In your results section, you simplify your presentation of results by saying "agreed" or "indicated" however your response scale is a 5-point likert-based scale. You do not describe a cut point in your scale that reflects "agree" or "disagree". Nor do your answer selection indicate a scale of agree to disagree. Some offer "never" to "extremely often" while others offer "not at all likely" to "extremely likely". I think stating "agreed" or "indicated" is somewhat misleading.**

Response: Thank you for your feedback and we have now provided clarity in the results section to more accurately describe our data. We have edited the results section to reflect responses from the 5-point Likert-based scale in the survey. Language throughout the body of the text was reviewed and updated to reflect responses and describe a cut point. The language in the abstract, specifically line 18, encompasses the cut point as suggested. The value was changed for accuracy. This change is also reflected in line 231.

6) **“I would have liked to have known whether your survey was pilot-tested or examined for psychometric properties such as reliability and validity.”**

Response: The purpose of the study was to pilot the survey at a single institute. This clarification has been added in line 81. Validation would require further testing, and this is addressed as a limitation in the manuscript.

7) **In your discussion, you make causal statements such as line 264 "The selectivity in VRS referral patterns is concerning." As your survey is descriptive in nature, I am not sure that level of causal effect is valid. Additionally, you also make this statement "A significant misconception among ECPs is that VRS represents an economic barrier for patients. In our study, 20% of respondents reported cost of devices was a barrier to patients receiving CRS". However, I am not sure asking the provider their opinion would accurately reflect this patient perspective as it was not the patient who was queried.**

Response: Thank you for your feedback and the section has now been revised to provide clarity in meaning. Line 264 has been revised to, “ECPs who participated in our survey demonstrate a pattern of selectivity when referring to VRS. Forty-two percent of ECPs reported location of services in relation to where the patient lives and stage of eye disease as a “very important” factor in their decision to refer to VRS” (Now starting at line 295).

In response to line 278, the cost of devices acts as surrogate for financial burden perception by a provider. Potentially, if a provider perceives the patient having limited finances or undergoing financial hardship, they may not refer to or discuss vision rehabilitation services.

8) **With respect to these statements, "Previous studies have consistently identified common factors affecting ECP referral decisions, including location of services, wait-times, availability, cost, patient inquiry about VRS, and driving considerations" & "Unfortunately, ECPs continue to wait for patients to initiate conversations about VRS before making referrals, most likely assuming they will receive negative reactions when broaching the topic I don't think it is as clear a pathway as to how your study confirms these patterns", can you please outline more clearly how your study confirms these patterns and overarching conclusions? Specifically, which questions and responses allow you to make these inferences?**

Response: Thank you for your feedback and we agree that this section needs further clarification. We have now provided specific data from our study that confirms these patterns. Please refer to lines 244-249 and 251-254 specifically. The following verbiage has been added, “Fifty-two percent of ECPs reported “very likely” and 23% reported “extremely likely” that a lack of VRS referrals were due to patients not inquiring about services. In terms of location of services to where the patient lives, 42% reported this as “very important” to their decision to refer to VRS. Regarding VRS availability and wait-times, 45% of ECPs reported this as “quite a bit” of a barrier for referring…Our results show 45% of ECPs assume it is “somewhat likely” patients will reject a referral to VRS. Fifty-two percent of ECPs assume a “somewhat” of a barrier to VRS is patient disinterest or lack of follow through with a referral while 26% felt this was “quite a bit” of a barrier.”

**Reviewer #2:**

1) **At times this didn't flow as well as I would have liked because there were so many new acronyms to me.**

Thank you for your review and recognizing the significance of our work. We have reviewed all acronyms used in the body of text. We have taken out the acronyms from the abstract for easier reading. Acronyms with definitions in the rest of the manuscript were revised to reflect those that were used most frequently to streamline the manuscript.

2) **You do touch on stigma, isolation, and lack of access to services, but I am missing a referral to structural ableism. In your discussion section about systemic barriers, you do a good job of getting at this, but I want a little more about why the ECPs lack awareness of resources. What kinds of bias are at play in these misconceptions? … Is this something you can expand on? Or if not, can you address this in the limitations section?**

Thank you for your comments. We did not specifically incorporate questions in our survey that would provide potential data of bias that would result in a lack of awareness of resources. For example, provider perception of the utility of these services could be tested. We have added this as a limitation in our manuscript. We posit that a lack of education is the main source of misconceptions about available resources, however, understanding why specialists, especially anterior segment specialists are not attending continuing education on low vision and rehabilitation would be helpful. In addition, reference to structural ableism is addressed in lines 48 and 377.

Again, thank you for your constructive feedback as well as your time in reviewing our manuscript. Please let us know if you have any further questions or need any more information.

Sincerely,

Sanbrita Mondal, OD

Kyle Peterson, PhD

Bonnie Verges, BS

Christina Thomas Virnig, PhD
