# Supplementary material for: Understanding decision making factors influencing referrals to vision rehabilitation services at a tertiary medical center
Source: PLoS One. 2026 Oct 26;21(10):e0359154. doi: 10.1371/journal.pone.0359154 (PMC13704866; doi:10.1371/journal.pone.0359154)
Supplement: Supplementary file 3 [file pone.0359154.s004.docx]

July 16, 2026

**Subject: Revision Submission – manuscript PONE-D-25-43062R1\**

**Dear Dr. Tucker,**

Thank you for the opportunity to submit our revised manuscript “Understanding decision making factors influencing referrals to vision rehabilitation services at a tertiary medical center” for consideration in PLOS ONE. We would like to extend our appreciation for the input and suggestions provided by both you and the reviewers. We have carefully considered all comments and have addressed them in our manuscript. We have provided an explanation for changes below. All line references refer to the unmarked revised “Manuscript”.

**Response to Editor:**

We have made all revisions to ensure that they align with the journal requirements and have now provided the following statement regarding consent from participants (line 103): “The information sheet for survey participation had language indicating implied consent.” Below is the information sheet highlighting language indicating implied consent:

**Thank you for your interest in our study.**

**Our goal is to understand provider perception of vision impairment and vision rehabilitation services. Your responses will enable us to recognize and address gaps to improve the process of vision rehabilitation and referrals for the overall psychosocial wellbeing of patients.**

**We invite you to complete this online questionnaire, which should take about 10 minutes to complete. All of your responses to this questionnaire are confidential. Any information you share will only be reported in group form and will not be reported in a way that would allow you to be personally identified.**

**If you have any questions about participating, please contact:**

**Sanbrita Mondal, OD.**

**Phone: 608-890-3945**

**Email: smondal4@wisc.edu**

**Instructions to complete the questionnaire:**

**To choose a response, click on the button that corresponds to your answer. If you would like to change your answer, click on a different button. You may go back to earlier screens by clicking the 'BACK' button at the bottom of the screen. To advance to the next screen click the 'NEXT' button.**

**If you start the questionnaire and are unable to finish it, you can exit by simply closing the browser window and return to it later by clicking again on the link in your email. This will take you back to the next unanswered question. If there is a question you leave blank, you will be informed that it was left blank. You may then either return to answer that question or continue on to the next question. You are not required to answer any question you would prefer to skip.**

**After the entire questionnaire has been completed and all the data are ready to be submitted, please click on the 'SUBMIT' button on the last screen. Once you click 'SUBMIT' you will not be able to re-enter the questionnaire.**

**We appreciate your time and effort in assisting us by completing this web-based questionnaire.**

**Please click next to begin the questionnaire now.**

**Reviewer #1:**

Thank you for taking the time to review our manuscript and for the feedback. We are glad all recommendations have been adequately addressed, and the manuscript reads more clearly.

Again, thank you for your feedback as well as your time in reviewing our manuscript. Please let us know if you have any further questions or need any more information.

Best regards,

Sanbrita Mondal, OD.
