# Supplementary material for: Understanding decision making factors influencing referrals to vision rehabilitation services at a tertiary medical center
Source: PLoS One. 2026 Oct 26;21(10):e0359154. doi: 10.1371/journal.pone.0359154 (PMC13704866; doi:10.1371/journal.pone.0359154)
Supplement: Peer review history

# Peer review: Decision Letter 0

**DOI:** 10.1371/journal.pone.0359154.r001

**Authors:** Clara Martínez Pérez

3 Jan 2026

Understanding decision making factors influencing referrals to vision rehabilitation services at a tertiary medical center

PLOS One

Dear Dr. MONDAL,

Thank you for submitting your manuscript to PLOS ONE. After careful consideration, we feel that it has merit but does not fully meet PLOS ONE’s publication criteria as it currently stands. Therefore, we invite you to submit a revised version of the manuscript that addresses the points raised during the review process.

Please submit your revised manuscript by Feb 17 2026 11:59PM. If you will need more time than this to complete your revisions, please reply to this message or contact the journal office at plosone@plos.org. When you're ready to submit your revision, log on to https://www.editorialmanager.com/pone/ and select the 'Submissions Needing Revision' folder to locate your manuscript file.

Please include the following items when submitting your revised manuscript:

- A letter that responds to each point raised by the academic editor and reviewer(s). You should upload this letter as a separate file labeled 'Response to Reviewers'.
- A marked-up copy of your manuscript that highlights changes made to the original version. You should upload this as a separate file labeled 'Revised Manuscript with Track Changes'.
- An unmarked version of your revised paper without tracked changes. You should upload this as a separate file labeled 'Manuscript'.

If you would like to make changes to your financial disclosure, please include your updated statement in your cover letter. Guidelines for resubmitting your figure files are available below the reviewer comments at the end of this letter.

If applicable, we recommend that you deposit your laboratory protocols in protocols.io to enhance the reproducibility of your results. Protocols.io assigns your protocol its own identifier (DOI) so that it can be cited independently in the future. For instructions see: https://journals.plos.org/plosone/s/submission-guidelines#loc-laboratory-protocols. Additionally, PLOS ONE offers an option for publishing peer-reviewed Lab Protocol articles, which describe protocols hosted on protocols.io. Read more information on sharing protocols at https://plos.org/protocols?utm_medium=editorial-email&utm_source=authorletters&utm_campaign=protocols.

We look forward to receiving your revised manuscript.

Kind regards,

Clara Martínez Pérez

Academic Editor

PLOS One

Journal Requirements:

When submitting your revision, we need you to address these additional requirements.

1.Please ensure that your manuscript meets PLOS ONE's style requirements, including those for file naming. The PLOS ONE style templates can be found at

https://journals.plos.org/plosone/s/file?id=wjVg/PLOSOne_formatting_sample_main_body.pdf and

https://journals.plos.org/plosone/s/file?id=ba62/PLOSOne_formatting_sample_title_authors_affiliations.pdf

2. Thank you for stating the following financial disclosure:

“This work was supported in part by an unrestricted grant from Research to Prevent Blindness, Inc. to the University of Wisconsin – Madison Department of Ophthalmology and Visual Sciences. This work was also supported by the UW-Madison Department of Ophthalmology and Visual Sciences Expanding our Vision Award.”

Please state what role the funders took in the study.  If the funders had no role, please state: "The funders had no role in study design, data collection and analysis, decision to publish, or preparation of the manuscript."

If this statement is not correct you must amend it as needed.

Please include this amended Role of Funder statement in your cover letter; we will change the online submission form on your behalf.

3. We note that you have indicated that there are restrictions to data sharing for this study. PLOS only allows data to be available upon request if there are legal or ethical restrictions on sharing data publicly. For more information on unacceptable data access restrictions, please see http://journals.plos.org/plosone/s/data-availability#loc-unacceptable-data-access-restrictions.

Before we proceed with your manuscript, please address the following prompts:

a) If there are ethical or legal restrictions on sharing a de-identified data set, please explain them in detail (e.g., data contain potentially identifying or sensitive patient information, data are owned by a third-party organization, etc.) and who has imposed them (e.g., a Research Ethics Committee or Institutional Review Board, etc.). Please also provide contact information for a data access committee, ethics committee, or other institutional body to which data requests may be sent.

b) If there are no restrictions, please upload the minimal anonymized data set necessary to replicate your study findings to a stable, public repository and provide us with the relevant URLs, DOIs, or accession numbers. For a list of recommended repositories, please see

https://journals.plos.org/plosone/s/recommended-repositories. You also have the option of uploading the data as Supporting Information files, but we would recommend depositing data directly to a data repository if possible.

We will update your Data Availability statement on your behalf to reflect the information you provide.

4. Please include your full ethics statement in the ‘Methods’ section of your manuscript file. In your statement, please include the full name of the IRB or ethics committee who approved or waived your study, as well as whether or not you obtained informed written or verbal consent. If consent was waived for your study, please include this information in your statement as well.

5. If the reviewer comments include a recommendation to cite specific previously published works, please review and evaluate these publications to determine whether they are relevant and should be cited. There is no requirement to cite these works unless the editor has indicated otherwise.

[Note: HTML markup is below. Please do not edit.]

Reviewer's Responses to Questions

**Comments to the Author**

1. Is the manuscript technically sound, and do the data support the conclusions?

Reviewer #1: Partly

Reviewer #2: Yes

**********

2. Has the statistical analysis been performed appropriately and rigorously?

Reviewer #1: Yes

Reviewer #2: I Don't Know

**********

3. Have the authors made all data underlying the findings in their manuscript fully available?

The PLOS Data policy

Reviewer #1: Yes

Reviewer #2: Yes

**********

4. Is the manuscript presented in an intelligible fashion and written in standard English?

Reviewer #1: No

Reviewer #2: Yes

**********

Reviewer #1: Thank you for the opportunity to review this manuscript on exploring the factors that influence ECP with VRS referral decision-making. The study employed survey research methodolgy to understand referral practices.

My first comment is there are several grammatical errors in the body of the text. The authors needs to better define their acronyms as some are used without definition (VI - only defined in the abstract), and some are used first and then defined later (AAO PPP). There are several sentences with grammatical errors. Here is one example, line 68 "Thus, it is important to identify barriers to care for and use that information to improve access to reach a greater population in need." line 100. This sentence is also awkward. "Data collected from all survey responses were accessed for analysis August 10, 2023." This sentence is really long and hard to read: line 122 "Analysis of our resulting survey data indicated that 76.9% (CI 50.7% - 92.2%) of 123 respondents with less than 10 YOP agreed patients should be referred to VRS at any visual acuity as directed by AAO PPP standard of care guidelines compared to 33.3% (CI 11.4% – 125 64.3%) of respondents with 10-19 YOP and to 44.4% (CI 18.5% - 73.4%) of respondents with 20 or more YOP (Fig 1).

I would have liked to have seen a table describing the demographic profile of the respondents (as described in lines102-105) and then broken up by their survey responses. You do not have that many respondents or survey questions and this would be easy to do.

You also state that "The psychosocial impact will be assessed in a separate report", however, I think your paper would benefit greatly if you discussed the psychosocial impact here in this paper together with your survey results".

You state that "Consent was not required by the Institutional Review Board as the study was not considered to involve human subjects" however your respondents are human and I see this as an error in judgment.

In your results section, you simplify your presentation of results by saying "agreed" or "indicated" however your response scale is a 5-point likert-based scale. You do not describe a cut point in your scale that reflects "agree" or "disagree". Nor do your answer selection indicate a scale of agree to disagree. Some offer "never" to "extremely often" while others offer "not at all likely" to "extremely likely". I think stating "agreed" or "indicated" is somewhat misleading.

Additionally, I would have liked to have known whether your survey was pilot-tested for examined for psychometric properties such as reliability and validity. Specifically, a survey that does not possess content validity would impact your results and interpretations.

In your discussion, you make causal statements such as line 264 "The selectivity in VRS referral patterns is concerning." As your survey is descriptive in nature, I am not sure that level of causal effect is valid. Additionally, you also make this statement "A significant misconception among ECPs is that VRS represents an economic barrier for

patients. In our study, 20% of respondents reported cost of devices was a barrier to patients

receiving CRS". However, I am not sure asking the provider their opinion would accurately reflect this patient perspective as it was not the patient who was queried.

With respect to these statements, "Previous studies have consistently identified common factors affecting ECP referral

decisions, including location of services, wait-times, availability, cost, patient inquiry about VRS, and driving considerations" & "Unfortunately, ECPs continue to wait for patients to initiate conversations about VRS before making referrals, most likely assuming they will receive negative reactions when broaching the topic I don't think it is as clear a pathway as to how your study confirms these patterns", can you please outline more clearly how your study confirms these patterns and overarching conclusions? Specifically, which questions and responses allow you to make these inferences? It is not clear to me after examining your survey.

Reviewer #2: You make a clear case for the importance of this research and the gap you are filling. A strength of the research is how you tie the data to your conclusions. I really appreciated the chance to review your work and understand more about what is happening with referrals to VCS.

At times this didn't flow as well as I would have liked because there were so many new acronyms to me. Is there any way to streamline these so a reader outside of your specialty does not have to keep so many in their head throughout?

While this is a paper about clinician referrals, I encourage you to reference Disability Studies whenever you're talking about a population that experiences disability. You do touch on stigma, isolation, and lack of access to services, but I am missing a reference to structural ableism. In your discussion section about systemic barriers, you do a good job of getting at this but I want a little more about why the ECPs lack awareness of resources. What kinds of bias are at play in these misconceptions? I'm obviously not saying all of these providers are horrible people, but there is a bias baked into perceived barriers that lead to fewer referrals. Is this something you can expand on? Or if not, can you address this in the limitations section?

**********

what does this mean?). If published, this will include your full peer review and any attached files.

If you choose “no”, your identity will remain anonymous but your review may still be made public.

**Do you want your identity to be public for this peer review?**  For information about this choice, including consent withdrawal, please see our Privacy Policy

Reviewer #1: **Yes:**  Dr. Breda Eubank

Reviewer #2: No

**********

[NOTE: If reviewer comments were submitted as an attachment file, they will be attached to this email and accessible via the submission site. Please log into your account, locate the manuscript record, and check for the action link "View Attachments". If this link does not appear, there are no attachment files.]

To ensure your figures meet our technical requirements, please review our figure guidelines: https://journals.plos.org/plosone/s/figures

You may also use PLOS’s free figure tool, NAAS, to help you prepare publication quality figures: https://journals.plos.org/plosone/s/figures#loc-tools-for-figure-preparation.

NAAS will assess whether your figures meet our technical requirements by comparing each figure against our figure specifications.

# Author comment: Author response to Decision Letter 1

**DOI:** 10.1371/journal.pone.0359154.r002

12 Feb 2026

Thank you for the opportunity to submit our revised manuscript “Understanding decision making factors influencing referrals to vision rehabilitation services at a tertiary medical center” for consideration in PLOS ONE. We would like to extend our appreciation for the input and suggestions provided by both you and the reviewers. We have carefully considered all comments, and the revisions have significantly improved our manuscript. We have provided an explanation of changes below. All line references refer to the unmarked revised “Manuscript.”

Response to Editor:

We have made all revisions to ensure that they align with the journal requirements and have now provided the following statement regarding the funder’s role “The funders had no role in study design, data collection and analysis, decision to publish, or preparation of the manuscript” in the financial disclosure and in the cover letter as requested. A de-identified data set is now shared. As requested, a full ethics statement has been added in the ‘Methods’ section in the manuscript file starting from line 98. In addition, although a laboratory protocol per se does not exist for this survey-based study, a copy of the survey was shared as Appendix 1. To honor the reviewers’ feedback regarding acronym use throughout the manuscript, we have condensed some of the less commonly used acronyms and have edited the titles and legends for Figures 1, 2, 4 and 5 to reflect these changes.

Reviewer #1:

1) “…there are several grammatical errors in the body of the text. The authors need to better define their acronyms as some are used without definition…”

Response: Thank you for taking the time to review our manuscript and for the detailed feedback. We have revised and corrected grammatical errors throughout the body of the text. As explained below, we have also modified the acronyms in the manuscript to facilitate flow and understanding.

All acronyms were revised and removed from the abstract. The acronyms VI, CE, ADL and VA were removed from the entire manuscript and only acronyms used most frequently remain in body of the text and are now defined including AAO PPP (line 49).

The specific sections provided in your review with grammatical errors have been revised and corrected. Line 68 has been revised and is now line 75. Line 100 has been revised and is now line 109. Line 122 has been revised and is now line 142.

2) “I would have liked to have seen a table describing the demographic profile of the respondents (as described in lines102-105) and then broken up by their survey responses.”

Response: Thank you for the suggestion. A table (see Table 1) and a description of the demographic profile of respondents have been added to the beginning of the results section (lines 132 to 137). In addition, differences in responses between genders were not found to be statistically significant for all results except for continuing education. This has been now added to the manuscript. Demographic profile is now added in the conclusion section.

3) You also state that "The psychosocial impact will be assessed in a separate report", however, I think your paper would benefit greatly if you discussed the psychosocial impact here in this paper together with your survey results".

Response: Thank you for your interest in the perceived patient psychosocial impact by providers. Psychosocial impact is an under reported issue that we believe will be best addressed in a separate report. We believe that sufficient data has been collected to justify a focused manuscript on this topic alone, rather than including it in our report on provider bias in referrals, where the findings could be diluted.

4) You state that "Consent was not required by the Institutional Review Board as the study was not considered to involve human subjects." The reviewer would like further clarification.

Response: Please refer to the IRB approval letter under ‘Others’ in the submission. This study went through full IRB review and was deemed as not research involving human subjects as defined by the Department of Health and Human Services and Food and Drug Administration regulations (Reference UW IRB ID# 2023-0119). Thus, obtaining consent for participation was not required. The letter does not provide any further information about the determination.

5) In your results section, you simplify your presentation of results by saying "agreed" or "indicated" however your response scale is a 5-point likert-based scale. You do not describe a cut point in your scale that reflects "agree" or "disagree". Nor do your answer selection indicate a scale of agree to disagree. Some offer "never" to "extremely often" while others offer "not at all likely" to "extremely likely". I think stating "agreed" or "indicated" is somewhat misleading.

Response: Thank you for your feedback and we have now provided clarity in the results section to more accurately describe our data. We have edited the results section to reflect responses from the 5-point Likert-based scale in the survey. Language throughout the body of the text was reviewed and updated to reflect responses and describe a cut point. The language in the abstract, specifically line 18, encompasses the cut point as suggested. The value was changed for accuracy. This change is also reflected in line 231.

6) “I would have liked to have known whether your survey was pilot-tested or examined for psychometric properties such as reliability and validity.”

Response: The purpose of the study was to pilot the survey at a single institute. This clarification has been added in line 81. Validation would require further testing, and this is addressed as a limitation in the manuscript.

7) In your discussion, you make causal statements such as line 264 "The selectivity in VRS referral patterns is concerning." As your survey is descriptive in nature, I am not sure that level of causal effect is valid. Additionally, you also make this statement "A significant misconception among ECPs is that VRS represents an economic barrier for patients. In our study, 20% of respondents reported cost of devices was a barrier to patients receiving CRS". However, I am not sure asking the provider their opinion would accurately reflect this patient perspective as it was not the patient who was queried.

Response: Thank you for your feedback and the section has now been revised to provide clarity in meaning. Line 264 has been revised to, “ECPs who participated in our survey demonstrate a pattern of selectivity when referring to VRS. Forty-two percent of ECPs reported location of services in relation to where the patient lives and stage of eye disease as a “very important” factor in their decision to refer to VRS” (Now starting at line 295).

In response to line 278, the cost of devices acts as surrogate for financial burden perception by a provider. Potentially, if a provider perceives the patient having limited finances or undergoing financial hardship, they may not refer to or discuss vision rehabilitation services.

8) With respect to these statements, "Previous studies have consistently identified common factors affecting ECP referral decisions, including location of services, wait-times, availability, cost, patient inquiry about VRS, and driving considerations" & "Unfortunately, ECPs continue to wait for patients to initiate conversations about VRS before making referrals, most likely assuming they will receive negative reactions when broaching the topic I don't think it is as clear a pathway as to how your study confirms these patterns", can you please outline more clearly how your study confirms these patterns and overarching conclusions? Specifically, which questions and responses allow you to make these inferences?

Response: Thank you for your feedback and we agree that this section needs further clarification. We have now provided specific data from our study that confirms these patterns. Please refer to lines 244-249 and 251-254 specifically. The following verbiage has been added, “Fifty-two percent of ECPs reported “very likely” and 23% reported “extremely likely” that a lack of VRS referrals were due to patients not inquiring about services. In terms of location of services to where the patient lives, 42% reported this as “very important” to their decision to refer to VRS. Regarding VRS availability and wait-times, 45% of ECPs reported this as “quite a bit” of a barrier for referring…Our results show 45% of ECPs assume it is “somewhat likely” patients will reject a referral to VRS. Fifty-two percent of ECPs assume a “somewhat” of a barrier to VRS is patient disinterest or lack of follow through with a referral while 26% felt this was “quite a bit” of a barrier.”

Reviewer #2:

1) At times this didn't flow as well as I would have liked because there were so many new acronyms to me.

Thank you for your review and recognizing the significance of our work. We have reviewed all acronyms used in the body of text. We have taken out the acronyms from the abstract for easier reading. Acronyms with definitions in the rest of the manuscript were revised to reflect those that were used most frequently to streamline the manuscript.

2) You do touch on stigma, isolation, and lack of access to services, but I am missing a referral to structural ableism. In your discussion section about systemic barriers, you do a good job of getting at this, but I want a little more about why the ECPs lack awareness of resources. What kinds of bias are at play in these misconceptions? … Is this something you can expand on? Or if not, can you address this in the limitations section?

Thank you for your comments. We did not specifically incorporate questions in our survey that would provide potential data of bias that would result in a lack of awareness of resources. For example, provider perception of the utility of these services could be tested. We have added this as a limitation in our manuscript. We posit that a lack of education is the main source of misconceptions about available resources, however, understanding why specialists, especially anterior segment specialists are not attending continuing education on low vision and rehabilitation would be helpful. In addition, reference to structural ableism is addressed in lines 48 and 377.

Attachment Submitted filename: PLOS Response To Reviewers 02.12.26.docx

# Peer review: Decision Letter 1

**DOI:** 10.1371/journal.pone.0359154.r003

**Authors:** Jennifer Tucker

6 Jul 2026

Dear Dr. MONDAL,

Thank you for submitting your manuscript to PLOS ONE. After careful consideration, we feel that it has merit but does not fully meet PLOS ONE’s publication criteria as it currently stands. Therefore, we invite you to submit a revised version of the manuscript that addresses the points raised during the review process.

Please submit your revised manuscript by Aug 20 2026 11:59PM. If you will need more time than this to complete your revisions, please reply to this message or contact the journal office at plosone@plos.org. When you're ready to submit your revision, log on to https://www.editorialmanager.com/pone/ and select the 'Submissions Needing Revision' folder to locate your manuscript file.

- A letter that responds to each point raised by the academic editor and reviewer(s). You should upload this letter as a separate file labeled 'Response to Reviewers'.
- A marked-up copy of your manuscript that highlights changes made to the original version. You should upload this as a separate file labeled 'Revised Manuscript with Track Changes'.
- An unmarked version of your revised paper without tracked changes. You should upload this as a separate file labeled 'Manuscript'.

If you would like to make changes to your financial disclosure, please include your updated statement in your cover letter. Guidelines for resubmitting your figure files are available below the reviewer comments at the end of this letter.

If applicable, we recommend that you deposit your laboratory protocols in protocols.io to enhance the reproducibility of your results. Protocols.io assigns your protocol its own identifier (DOI) so that it can be cited independently in the future. For instructions see: https://journals.plos.org/plosone/s/submission-guidelines#loc-laboratory-protocols. Additionally, PLOS ONE offers an option for publishing peer-reviewed Lab Protocol articles, which describe protocols hosted on protocols.io. Read more information on sharing protocols at https://plos.org/protocols?utm_medium=editorial-email&utm_source=authorletters&utm_campaign=protocols.

As the corresponding author, your ORCID iD is verified in the submission system and will appear in the published article. PLOS supports the use of ORCID, and we encourage all coauthors to register for an ORCID iD and use it as well. Please encourage your coauthors to verify their ORCID iD within the submission system before final acceptance, as unverified ORCID iDs will not appear in the published article. *Only*  the individual author can complete the verification step; PLOS staff *cannot*  verify ORCID iDs on behalf of authors.

We look forward to receiving your revised manuscript.

Kind regards,

Jennifer Tucker, PhD

Staff Editor

PLOS One

Journal Requirements:

1. If the reviewer comments include a recommendation to cite specific previously published works, please review and evaluate these publications to determine whether they are relevant and should be cited. There is no requirement to cite these works unless the editor has indicated otherwise.

2. Please review your reference list to ensure that it is complete and correct. If you have cited papers that have been retracted, please include the rationale for doing so in the manuscript text, or remove these references and replace them with relevant current references. Any changes to the reference list should be mentioned in the rebuttal letter that accompanies your revised manuscript. If you need to cite a retracted article, indicate the article’s retracted status in the References list and also include a citation and full reference for the retraction notice.

[Note: HTML markup is below. Please do not edit.]

Reviewers' comments:

Reviewer's Responses to Questions

**Comments to the Author**

Reviewer #1: All comments have been addressed

**********

2. Is the manuscript technically sound, and do the data support the conclusions?

Reviewer #1: Yes

**********

3. Has the statistical analysis been performed appropriately and rigorously?

Reviewer #1: Yes

**********

4. Have the authors made all data underlying the findings in their manuscript fully available?

The PLOS Data policy

Reviewer #1: Yes

**********

5. Is the manuscript presented in an intelligible fashion and written in standard English?

Reviewer #1: Yes

**********

Reviewer #1: Thank you for. the opportunity to re-review your manuscript. All recommendations have been adequately addressed. Your manuscript reads clearer.

**********

what does this mean?). If published, this will include your full peer review and any attached files.

If you choose “no”, your identity will remain anonymous but your review may still be made public.

**Do you want your identity to be public for this peer review?**  For information about this choice, including consent withdrawal, please see our Privacy Policy

Reviewer #1: **Yes:**  Breda Eubank

**********

[NOTE: If reviewer comments were submitted as an attachment file, they will be attached to this email and accessible via the submission site. Please log into your account, locate the manuscript record, and check for the action link "View Attachments". If this link does not appear, there are no attachment files.]

To ensure your figures meet our technical requirements, please review our figure guidelines: https://journals.plos.org/plosone/s/figures

You may also use PLOS’s free figure tool, NAAS, to help you prepare publication quality figures: https://journals.plos.org/plosone/s/figures#loc-tools-for-figure-preparation.

NAAS will assess whether your figures meet our technical requirements by comparing each figure against our figure specifications.

# Author comment: Author response to Decision Letter 2

**DOI:** 10.1371/journal.pone.0359154.r004

16 Jul 2026

Thank you for the opportunity to submit our revised manuscript “Understanding decision making factors influencing referrals to vision rehabilitation services at a tertiary medical center” for consideration in PLOS ONE. We would like to extend our appreciation for the input and suggestions provided by both you and the reviewers. We have carefully considered all comments and have addressed them in our manuscript. We have provided an explanation for changes below. All line references refer to the unmarked revised “Manuscript”.

Response to Editor:

We have made all revisions to ensure that they align with the journal requirements and have now provided the following statement regarding consent from participants (line 103): “The information sheet for survey participation had language indicating implied consent.” Below is the information sheet highlighting language indicating implied consent:

Thank you for your interest in our study.

Our goal is to understand provider perception of vision impairment and vision rehabilitation services. Your responses will enable us to recognize and address gaps to improve the process of vision rehabilitation and referrals for the overall psychosocial wellbeing of patients.

We invite you to complete this online questionnaire, which should take about 10 minutes to complete. All of your responses to this questionnaire are confidential. Any information you share will only be reported in group form and will not be reported in a way that would allow you to be personally identified.

If you have any questions about participating, please contact:

Sanbrita Mondal, OD.

Phone: 608-890-3945

Email: smondal4@wisc.edu

Instructions to complete the questionnaire:

To choose a response, click on the button that corresponds to your answer. If you would like to change your answer, click on a different button. You may go back to earlier screens by clicking the 'BACK' button at the bottom of the screen. To advance to the next screen click the 'NEXT' button.

If you start the questionnaire and are unable to finish it, you can exit by simply closing the browser window and return to it later by clicking again on the link in your email. This will take you back to the next unanswered question. If there is a question you leave blank, you will be informed that it was left blank. You may then either return to answer that question or continue on to the next question. You are not required to answer any question you would prefer to skip.

After the entire questionnaire has been completed and all the data are ready to be submitted, please click on the 'SUBMIT' button on the last screen. Once you click 'SUBMIT' you will not be able to re-enter the questionnaire.

We appreciate your time and effort in assisting us by completing this web-based questionnaire.

Please click next to begin the questionnaire now.

Reviewer #1:

Thank you for taking the time to review our manuscript and for the feedback. We are glad all recommendations have been adequately addressed, and the manuscript reads more clearly.

Again, thank you for your feedback as well as your time in reviewing our manuscript. Please let us know if you have any further questions or need any more information.

Attachment Submitted filename: Response to Reviewers 07.16.2026.docx

# Editor report: Decision Letter 2

**DOI:** 10.1371/journal.pone.0359154.r005

**Authors:** Jennifer Tucker

10 Sep 2026

Understanding decision making factors influencing referrals to vision rehabilitation services at a tertiary medical center

PONE-D-25-43062R2

Dear Dr. MONDAL,

We’re pleased to inform you that your manuscript has been judged scientifically suitable for publication and will be formally accepted for publication once it meets all outstanding technical requirements.

Within one week, you’ll receive an e-mail detailing the required amendments. When these have been addressed, you’ll receive a formal acceptance letter and your manuscript will be scheduled for publication.

An invoice will be generated when your article is formally accepted. Please note, if your institution has a publishing partnership with PLOS and your article meets the relevant criteria, all or part of your publication costs will be covered. Please make sure your user information is up-to-date by logging into Editorial Manager at Editorial Manager® and clicking the ‘Update My Information' link at the top of the page. For questions related to billing, please contact billing support.

If your institution or institutions have a press office, please notify them about your upcoming paper to help maximize its impact. If they’ll be preparing press materials, please inform our press team as soon as possible -- no later than 48 hours after receiving the formal acceptance. Your manuscript will remain under strict press embargo until 2 pm Eastern Time on the date of publication. For more information, please contact onepress@plos.org.

Kind regards,

Jennifer Tucker, PhD

Staff Editor

PLOS One

Additional Editor Comments (optional):

Reviewers' comments:

# Editor report: Acceptance letter

**DOI:** 10.1371/journal.pone.0359154.r006

**Authors:** Jennifer Tucker

PONE-D-25-43062R2

PLOS One

Dear Dr. MONDAL,

I'm pleased to inform you that your manuscript has been deemed suitable for publication in PLOS One. Congratulations! Your manuscript is now being handed over to our production team.

At this stage, our production department will prepare your paper for publication. This includes ensuring the following:

* All references, tables, and figures are properly cited

* All relevant supporting information is included in the manuscript submission,

* There are no issues that prevent the paper from being properly typeset

You will receive further instructions from the production team, including instructions on how to review your proof when it is ready. Please keep in mind that we are working through a large volume of accepted articles, so please give us a few days to review your paper and let you know the next and final steps.

Lastly, if your institution or institutions have a press office, please let them know about your upcoming paper now to help maximize its impact. If they'll be preparing press materials, please inform our press team within the next 48 hours. Your manuscript will remain under strict press embargo until 2 pm Eastern Time on the date of publication. For more information, please contact onepress@plos.org.

You will receive an invoice from PLOS for your publication fee after your manuscript has reached the completed accept phase. If you receive an email requesting payment before acceptance or for any other service, this may be a phishing scheme. Learn how to identify phishing emails and protect your accounts at https://explore.plos.org/phishing.

If we can help with anything else, please email us at customercare@plos.org.

Thank you for submitting your work to PLOS One and supporting open access.

Kind regards,

PLOS One Editorial Office Staff

on behalf of

Dr Jennifer Tucker

Staff Editor

PLOS One
